# Opioid agonist and antagonist use and the gut microbiota: associations among people in addiction treatment

**DOI:** 10.1038/s41598-020-76570-9

**Published:** 2020-11-10

**Authors:** Rachel E. Gicquelais, Amy S. B. Bohnert, Laura Thomas, Betsy Foxman

**Affiliations:** 1 University of Wisconsin-Madison School of Nursing, 701 Highland Avenue, Madison, WI 53705 USA; 2 Department of Epidemiology, Johns Hopkins Bloomberg School of Public Health, 615 N. Wolfe St, Baltimore, MD 21205 USA; 3 Department of Epidemiology, University of Michigan School of Public Health, 1415 Washington Heights, Ann Arbor, MI 48109 USA; 4 Department of Psychiatry, University of Michigan Medical School, 2800 Plymouth Rd, Ann Arbor, MI 48109 USA; 5 VA Center for Clinical Management Research, 2800 Plymouth Rd, Ann Arbor, MI 48109 USA; 6 Department of Anesthesiology, University of Michigan Medical School, Ann Arbor, 48109 USA; 7 Department of Epidemiology, University of Michigan Medical School, 1415 Washington Heights, Ann Arbor, MI 48109 USA

**Keywords:** Addiction, Epidemiology

## Abstract

Murine models suggest that opioids alter the gut microbiota, which may impact opioid tolerance and psychopathology. We examined how gut microbiota characteristics related to use of opioid agonists and antagonists among people receiving outpatient addiction treatment. Patients (n = 46) collected stool samples and were grouped by use of opioid agonists (heroin, prescription opioids), antagonists (naltrexone), agonist–antagonist combinations (buprenorphine–naloxone), or neither agonists nor antagonists within the month before enrollment. We sequenced the V4 region of the 16S rRNA gene using Illumina MiSeq to examine how alpha diversity, enterotypes, and relative abundance of bacterial genera varied by opioid agonist and antagonist exposures. Compared to 31 participants who used neither agonists nor antagonists, 5 participants who used opioid agonists (without antagonists) had lower microbiota diversity, *Bacteroides* enterotypes, and lower relative abundance of *Roseburia*, a butyrate producing genus, and *Bilophila*, a bile acid metabolizing genus. There were no differences in gut microbiota features between those using agonist + antagonists (n = 4), antagonists only (n = 6), and neither agonists nor antagonists. Similar to murine morphine exposure models, opioid agonist use was associated with lower microbiota diversity. Lower abundance of *Roseburia* and *Bilophila* may relate to the gut inflammation/permeability and dysregulated bile acid metabolism observed in opioid-exposed mice.

## Introduction

Morbidity and mortality related to opioid use have increased dramatically during the past two decades^1^. There is an urgent need to improve opioid use disorder (OUD) treatment outcomes to mitigate this growing morbidity and mortality^2^. One relatively unexplored source of potential markers for OUD treatment effects and OUD etiology are those from the gut microbiota, which influences signaling along the gut-brain axis^3–6^. Murine models have identified several rapid gut microbiota changes associated with morphine exposure, including decreased richness (i.e., number of bacterial species) and increased abundance of *Enterococcus faecalis* compared to placebo-treated mice^7^. Morphine, an opioid agonist, caused several other negative consequences to gut health in mice, including increased intestinal permeability (i.e., gut leakiness), heightened infection risk, bacterial translocation to mesenteric lymph nodes and the liver, dysregulated immune responses, disruptions to bile acid metabolism, inflammation, and induction of virulence factor expression in pathogenic bacteria^7–11^.

The relationship between gut health and OUD etiology and recovery has only begun to be characterized, and few studies have examined these relationships in humans^12–14^. Murine models suggest that alterations to the gut microbiota related to diminished gut motility from the constipating effects of opioids, gut barrier disruption, and local and systemic inflammation may modulate the development of opioid tolerance, the need for higher doses of opioids to attain equivalent antinociceptive or euphoric effects over time^6, 11^. Because tolerance to the antinociceptive effects of opioids may develop faster than tolerance to the respiratory depressive effects of opioids, the relationship of the gut microbiota with tolerance may importantly impact overdose risk^6,15^.

Additionally, a handful of studies on chronic alcohol use suggest the importance of healthy gut barrier function to recovery from alcohol use disorders^5,16–18^. Stärkel et al*.* hypothesize that gut leakiness from chronic drinking and the release of bacterial products into the bloodstream could promote a neuro-inflammatory state that influences mood and drinking behaviors^5^. In the context of OUD, a murine model suggests that dietary supplementation with anti-inflammatory omega-3 polyunsaturated fatty acids alter the gut microbiota and reduce anxiety symptoms and opioid-seeking behaviors^19^.

In addition to omega-3 polyunsaturated fatty acids^19^, some of the negative consequences associated with opioid exposure may be preventable by co-treatment with probiotics^11^ and opioid antagonists, such as naltrexone^7,8^. For example, mice co-treated with morphine and naltrexone do not exhibit the increases in *E. faecalis* or disruptions to bile acid metabolism observed in morphine treated mice^7^. This preliminary evidence for the antagonism of opioid effects on the gut suggests that alterations associated with opioid use relate to the binding of opioid agonists to μ-opioid receptors^7,8^. That probiotic treatment attenuates the development of morphine tolerance in mice further implicates the importance of the gut microbiota in OUD^11^.

Two types of opioid antagonists, which block the binding of opioid agonists (e.g., heroin and prescription opioids) to the μ-opioid receptor, are used in OUD treatment^20^. Naltrexone is used to manage cravings for both alcohol and OUDs^20^. It is also used to relieve opioid-induced constipation and is thought to mitigate gut mucosal injury from Crohn’s disease, suggesting that it may modulate gut barrier function^21–25^. It is unknown whether the formulation of naltrexone used to manage cravings for opioid and alcohol use disorders similarly modulates gut barrier function and/or whether this modulation of gut barrier function has a possible role in craving management. A second clinically relevant opioid antagonist is naloxone. Alone, naloxone reverses the respiratory depressive effects from high doses of opioids that occur during an opioid overdose. In combination with buprenorphine, a partial opioid agonist, buprenorphine–naloxone is one of the most commonly used medications for OUDs^20,26^. Naloxone is included in buprenorphine formulations to reduce the risk of abuse^26^. Although the bioavailability of oral naloxone in common formulations of buprenorphine–naloxone is low^27^, it is unknown how the small amounts metabolized during daily dosing impact the gut microbiota or how buprenorphine, a partial opioid agonist^26^, impacts the gut microbiota differently from full agonists.

While opioid agonist exposure disrupts the gut microbiota in mice^7–9^, this process has not been well described in humans who use opioids^13,14^. Further, no studies have evaluated whether the effects of opioids on the microbiota are observed in patients taking opioid antagonists, such as naltrexone, or agonist–antagonist combinations (e.g., buprenorphine–naloxone). To gain insight into the impacts on humans, we examined the relationship between opioid agonist and antagonist exposures and characteristics of the gut microbiota among 46 patients receiving outpatient addiction treatment. Based on murine models, we hypothesized that opioid agonist use would be associated with decreased microbiota alpha diversity and that participants who used agonist–antagonist combinations would have gut microbiota features more similar to those who used neither agonists nor antagonists.

## Methods

### Participant recruitment

We recruited participants from the patient population attending a private, outpatient addiction treatment facility in Michigan during July 2016 through September 2017. The facility offered medications for OUD, including buprenorphine–naloxone and naltrexone, but did not offer methadone. Research assistants approached patients to assess eligibility and obtain consent. Eligible patients had to be ≥ 18 years, speak English, be able to provide informed consent, and be able to see, speak, and hear. We excluded people who were unable to provide informed consent for any reason, including acute intoxication or insufficient cognitive functioning (indicated by Mini-Mental State Exam score < 21)^28^. Participants provided written informed consent and completed a 45-min survey. A total of 124 participants provided informed consent and 92 (74.2%) completed the initial survey within 4 weeks (Supplementary Fig. S1). We compensated participants $5.

We invited 65 participants to enroll in a microbiota study. Eligibility criteria for the microbiota study included age 18–60 years and self-reported use of at least 1 substance in the past 30 days or misuse of prescription opioids during the month before beginning treatment (further described in Supplementary Methods). We enrolled 51 participants, who completed an additional survey about their dietary habits and were compensated $20 (Supplementary Fig. S1). Weekly thereafter for three weeks, participants submitted a stool sample and completed a survey reporting their depression, anxiety, cravings to use drugs or alcohol, dietary habits, and antibiotic use. We compensated participants $10 per appointment. All appointments were completed within one month of enrollment in the microbiota study. This analysis includes data from 46 participants who provided at least one stool sample.

As part of their written informed consent, participants granted the researchers access to their medical record. We reviewed medical encounters during the 30 days before completion of the first survey through the day of sample collection and noted prescriptions for naltrexone and buprenorphine–naloxone using University of Michigan’s Electronic Medical Record Search Engine followed by medical record review to confirm medication use^29^. The study was approved by the Institutional Review Board at the University of Michigan (HUM00113964) and all methods were performed in accordance with relevant guidelines and regulations.

### Measures

We collapsed opioid agonist/antagonist use into four categories: agonist only (Ag), combined agonist–antagonist (AgAt), antagonist only (At), or neither opioid agonist nor antagonist (N). Participants in the Ag group self-reported opioid use (heroin or prescription opioids used as prescribed or not as prescribed) in the 30 days before study enrollment on the substance use survey and did not have a documented prescription for buprenorphine–naloxone or naltrexone in the medical record at the time of sample collection. Participants with combined AgAt use either (1) self-reported opioid use and had a prescription for naltrexone documented in their medical record during the time of sample collection, or (2) were prescribed buprenorphine–naloxone during the time of sample collection. Participants in the At group had a prescription for naltrexone documented in their medical record during the day of sample collection (no participants were prescribed a supply of standalone naloxone per the medical record). Finally, participants in the N group self-reported neither opioid use nor had a prescription for naltrexone or buprenorphine–naloxone in the medical record during the time of sample collection. Self-reported opioid use was assessed using the Alcohol, Smoking and Substance Involvement Screening Test (ASSIST)^30^. We used a validated dietary screener to quantify dietary fiber intake during the week before sample collection^31^. Participants additionally self-reported alcohol use during the 30 days before the substance use survey using the ASSIST^30^. A complete summary of measures is provided in the Supplementary Methods.

### Sequencing

Participants self-collected stool samples using previously described protocols^32–34^. Briefly, participants placed two dime-sized scoops of stool into a sterile Sarstedt tube with a spoon lid (Sarstedt, Nümbrecht, Germany) containing a cryopreservant, RNA*later*™ (Ambion, Austin, TX), and 5–10 glass beads (Walter Stern, Washington, NY). RNA*later*™ was used to maintain the stool microbial community composition under room temperature conditions for several days^33,34^, allowing participants flexibility in the timing of sample collection relative to study appointments. Participants homogenized samples by shaking and samples were frozen at -80C within two days of collection. Deoxyribonucleic acid (DNA) extraction and Illumina MiSeq sequencing of the V4 hypervariable region of the bacterial 16S ribosomal ribonucleic acid (rRNA) gene was completed using previously published protocols^35–37^.

We processed sequencing reads using mothur (v1.39.5) and the MiSeq standard operating procedure (https://www.mothur.org/wiki/MiSeq_SOP, accessed November 8, 2017)^37^. We clustered samples into oligotypes using previously described procedures^38,39^. We removed samples with < 1000 reads and verified that mock communities resembled their known compositions. We assigned oligotype taxonomy using the Ribosomal Database Project (RDP, release 11, update 5)^40^.

In the main analysis, we examined the first sample submitted per participant, amounting to 46 samples with 2,207,827 sequence reads (21,796–77,013 reads per participant) and 354 oligotypes. The first sample was selected for the main analysis as this provided the most proximal assessment of the stool microbiota to the ASSIST. In sensitivity analyses, we examined the second and third samples submitted per participant to assess consistency with main findings from the first submitted sample.

### Microbiota measures

We calculated the relative abundance of genera in each sample (i.e., the number of sequencing reads from each genus divided by the total number of sequencing reads per sample). We summarized alpha diversity using Shannon diversity, a measure of the number and evenness of oligotypes, and the Chao1 Index, a measure of microbiota richness based on the number of oligotypes. Alpha diversity analyses were completed after rarefaction^41^ of sequencing depth to 90% of the maximum sequencing depth as recommended by Knight et al.(see Supplementary Methods for further details)^42^. As a sensitivity analysis, we also examined results without rarefaction, as recommended by McMurdie and Holmes^43^. We visualized between-sample differences (beta diversity) using principal component analysis of Aitchison distance^44^.

We next classified each sample’s genus-level read counts into enterotypes (i.e., bacterial community types) using two clustering techniques^45,46^. Enterotyping methods cluster samples with similar taxa distributions into discrete groups using the taxa covariance matrix^45–47^. First, we used Dirichlet multinomial mixture (DMM) models to assign de novo enterotypes based on our samples^45^. We identified the number of enterotypes that minimized the Laplace approximation of negative log models for DMM models with one to five enterotypes^45^. We assigned each sample to its most likely enterotype based on posterior probabilities of enterotype assignment (minimum posterior probability: 95.3%). To describe the bacterial profiles typical of each enterotype, we summarized the genera distribution for each enterotype and examined alpha diversity by enterotype.

Secondarily, we assigned each sample to one of three previously described enterotypes observed in healthy humans by uploading our genus-level relative abundance data to https://enterotypes.org^46^. This assigned each sample to one of three reference-based enterotypes, each dominated by *Bacteroides*, Firmicutes, or *Prevotella,* by comparing our data to that from the Human Microbiome Project (HMP) and Metagenomics of the Human Intestinal Tract (MetaHIT). Two of 46 samples from the first study visit were not comparable to reference samples and were assigned as “missing.” See the Supplementary Methods for further explanation of the enterotyping methods used.

### Statistical analysis

We compared microbiota diversity, enterotypes, and genera relative abundance among participants exposed to opioid agonists (Ag), agonist–antagonist combinations (AgAt), or antagonists alone (At) to participants exposed to neither opioid agonists nor antagonists (N). We compared alpha diversity metrics (Shannon and Chao1) using Wilcoxon rank sum tests. We visualized beta diversity using principal component analyses of Aitchison distance. We compared the distribution of de novo enterotypes with Fisher’s exact test.

We identified bacterial genera that were differentially abundant between opioid agonist–antagonist use groups using ALDEx2, an analysis of variance-like tool for microbiota data^48^. Like analysis of variance, ALDEx2 identifies genera with greater between than within group differences. We implemented a nonparametric version of ALDEx2 that compared genera centered log ratios using the Wilcoxon rank sum test and set an a priori statistical significance threshold for the false discovery rate corrected *p* value < 0.05 (using the Benjamini–Hochberg procedure). See the Supplementary Methods for further details on ALDEx2.

We summarized differences in dietary fiber intake and self-reported past 30-day alcohol use by both microbiota characteristics (diversity, enterotypes, and genera differential abundance) and opioid agonist–antagonist use. These variables were explored as potential alternative explanations for our findings around opioid use given previously documented associations of these characteristics with the gut microbiota^16,49–51^.

## Results

The median age of the 46 participants was 33.5 years; roughly half were male (56.5%), and most were white (84.7%) and Non-Hispanic (89.1%, Table 1). Five used opioid agonists only (heroin or prescription opioids, Ag), 4 used agonists and antagonists (AgAt, 3 buprenorphine–naloxone and 1 prescription opioids and naltrexone), 6 used an opioid antagonist only (At, naltrexone), and 31 used neither opioid agonists nor antagonists (N, Supplementary Fig. S1).

**Table 1:** Characteristics of 46 Study Participants Enrolled from an Outpatient Addiction Treatment Facility, 2016–2017.

Characteristic	Total n (%)	Ag n (%)	AgAt n (%)	At n (%)	N n (%)
Total	46 (100)	5 (100)	4 (100)	6 (100)	31 (100)
Age, median (IQR)	33.5 (26.3–47.5)	38 (31–46)	27.5 (23.5–35.8)	34 (27.5–44.3)	36 (25.5–48)
**Gender**					
Female	26 (41.3)	1 (20.0)	2 (50.0)	1 (16.7)	15 (48.4)
Male	19 (56.5)	4 (80.0)	2 (50.0)	5 (83.3)	15 (48.4)
Other	1 (2.2)	0 (0)	0 (0)	0 (0)	1 (3.2)
**Race**					
Black	2 (4.3)	0 (0)	0 (0)	0 (0)	2 (6.5)
White	39 (84.7)	3 (60.0)	4 (100.0)	5 (83.3)	27 (87.1)
Multiple Races	2 (4.3)	1 (20.0)	0 (0)	0 (0)	1 (3.2)
Other	3 (6.5)	1 (20.0)	0 (0)	1 (16.7)	1 (3.2)
Hispanic ethnicity	5 (10.9)	1 (20.0)	0 (0)	1 (16.7)	3 (9.7)
Used alcohol^a^	34 (73.9)	3 (60.0)	3 (75.0)	5 (83.3)	23 (74.2)
Antibiotic use	1 (2.2)	0 (0)	1 (25.0)	0 (0)	0 (0)
Days in treatment, Median (IQR)	34 (5–74)	12 (3–1171)	549 (123–949)	23 (6–53)	19 (6–67)
Fiber (g/day), median (IQR)^b^	15.7 (14.1–18.4)	13.5 (12.7–17.0)	17.9 (14.9–19.0)	16.9 (13.2–18.3)	15.6 (14.3–17.7)
Depression Score, median (IQR)^c^	9.5 (6.0–12.8)	13 (12–18)	7 (6.5–8.8)	9 (6.3–11)	9 (5.5–11.5)
Anxiety Score, median (IQR)^d^	8 (4–10)	13 (9–14)	4.5 (1.5–8)	8 (7–9.8)	7 (3–9.5)
Craving Score, median (IQR)^e^	9 (5–16)	16 (9–19)	5.5 (6.5–13)	12 (6.8–17.3)	8.5 (5–13.8)

*Ag* opioid agonist only (heroin [n = 2] or prescription opioid [n = 3]), *AgAt* opioid agonist–antagonist use (buprenorphine–naloxone [n = 3] or prescription opioids + naltrexone [n = 1]), *At* opioid antagonist use only (naltrexone [n = 6]), *N* neither opioid agonist nor antagonist use (n = 31), *IQR* interquartile range.

^a^Participants self-reported alcohol use in the 30 days before the substance use survey (before enrolling in the microbiota study).

^b^Fiber intake data were available for 45 of 46 participants (30 of 31 participants who used neither opioid agonists nor antagonists).

^c^Score from the Patient Health Questionnaire (PHQ)-9 (range 0–27).

^d^Score from the Generalized Anxiety Disorder 7-Item scale (range 0–21).

^e^Score from the modified Penn Craving Scale (range 0–30). Data were available for 45 of 46 participants (30 of 31 participants who used neither opioid agonists not antagonists).

### Microbiota diversity

**Figure 1:**
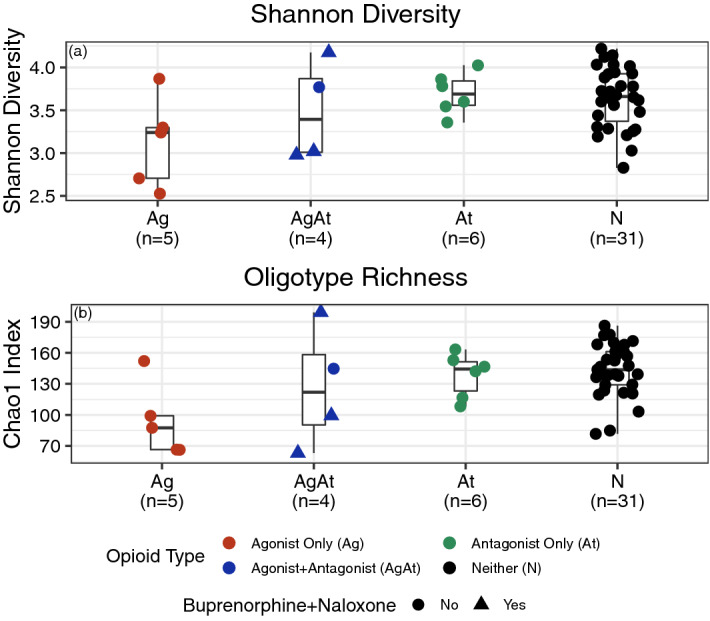
Gut microbiota alpha diversity among 46 participants receiving outpatient addiction treatment, 2016–2017. We compared alpha diversity between opioid agonist only (Ag), agonist + antagonist (AgAt), and antagonist only (At) vs. neither agonist nor antagonist (N) groups using two metrics. Ag participants had lower diversity compared to N for both Shannon diversity (**a**, Wilcoxon rank sum *p* = 0.04) and richness (**b**, Chao1 index, *p* = 0.008). No other groups statistically differed, including AgAt vs. N and At vs. N.

Ag participants had lower Shannon diversity (*p* = 0.04) and richness (*p* = 0.008) vs. N participants (Fig. 1). Shannon diversity and richness for AgAt and At participants did not statistically differ from N participants. These differences remained in sensitivity analyses without rarefaction (data not shown) and in the second and third stool samples submitted per participant (Supplemental Table S1), with the exception that Shannon Diversity between Ag vs. N participants was only marginally different (*p* = 0.05 and *p* = 0.08 in samples two and three, respectively). The consistency in findings across samples collected over time likely related to the relative stability in alpha diversity (Supplementary Fig. S2). Plots of a principal component analysis of Aitchison distance showed no clustering by opioid agonist–antagonist groups (Supplementary Fig. S3), which was similar across the second and third samples (Supplementary Table S1). Like alpha diversity, beta diversity also suggested relative consistency across samples from the same participant over time (Supplementary Fig. S4).

### De novo assigned enterotypes

We identified three enterotypes using Dirichlet multinomial mixture models: two *Bacteroides* dominated groups and a third dominated by *Prevotella* (23.9% of participants, n = 11, Supplementary Figs. S5, S6a, and S6b). Among the 35 participants with *Bacteroides* dominated groups, 24 had elevated *Faecalibacterium* (*Bacteroides: Faec.*) and 11 had elevated *Clostridium* cluster XIVa (*Bacteroides: Clost.*)*.*

**Figure 2:**
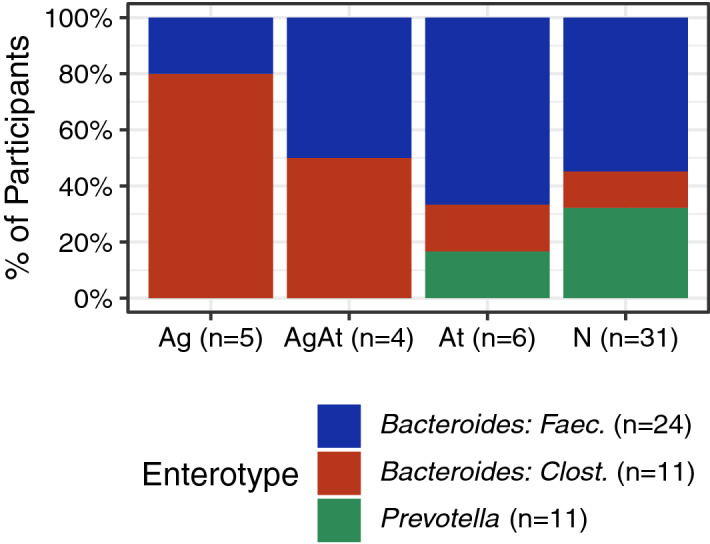
De novo assigned gut microbiota enterotypes among 46 participants receiving outpatient addiction treatment, 2016–2017. The prevalence of three enterotypes identified through Dirichlet multinomial mixture modeling differed by opioid agonist–antagonist exposure groups. No individuals who used opioid agonists (agonist only [Ag] nor agonist–antagonist combination [AgAt]) had the *Prevotella* enterotype and 4 of 5 Ag participants had a *Bacteroides* enterotype with elevated *Clostridium* cluster XIVa. The distribution of enterotypes differed between Ag participants vs. participants who used neither agonists nor antagonists (N, Fisher exact *p* value = 0.006). *Ag* agonist only, *AgAt* agonist–antagonist, *At* antagonist only, *N* neither agonist nor antagonist.

No Ag or AgAt participants had the *Prevotella* enterotype (Fig. 2). The distribution of enterotypes differed between Ag and N groups; *Bacteroides: Clost.* was more common in the Ag group (Fisher exact *p* value = 0.006). The *Bacteroides: Clost.* group had lower alpha diversity (Supplementary Fig. S7). We did not detect other statistically significant differences in the distribution of enterotypes (i.e., AgAt vs. N and At vs. N). These trends were similar across the second and third samples (Supplemental Table S1).

### De novo versus reference-based enterotypes from healthy human studies

The majority of samples (95.7%, 44/46) were similar enough to the genera distributions observed in healthy human populations to be assigned a reference-based enterotype (Table 2). The enterotype assignments for 42 of 44 samples were the same using the de novo and reference-based methods. Regardless of method, most samples were assigned the *Bacteroides* enterotype. No participants assigned the *Prevotella* enterotype used opioid agonists (Ag or AgAt). Both samples that were unable to be assigned to a reference-based enterotype were assigned the *Bacteroides: Clost. *de novo enterotype. These results were consistent across the second and third samples (Supplemental Table S1).

**Table 2:** Reference-based and de novo enterotypes among 46 participants receiving outpatient addiction treatment, 2016–2017.

Reference-based enterotypes (Method: PAM)	De novo assigned enterotypes (Method: DMM)			
	*Bacteroides: Faec* n (%)	*Bacteroides: Clost* n (%)	*Prevotella* n (%)	Total n (%)
*Bacteroides*	22 (91.7)	9 (81.8)	0 (0)	31 (67.4)
Firmicutes	0 (0)	0 (0)	0 (0)	0 (0)
*Prevotella*	2 (8.3)	0 (0)	11 (100)	13 (28.3)
Unable to be assigned	0 (0)	2 (18.2)	0 (0)	2 (4.3)
Total	24 (100)	11 (100)	11 (100)	46 (100)

*DMM* Dirichlet multinomial mixture model, *PAM* partitioning around medoid clustering, *n* number.

### Differentially abundant genera

**Figure 3:**
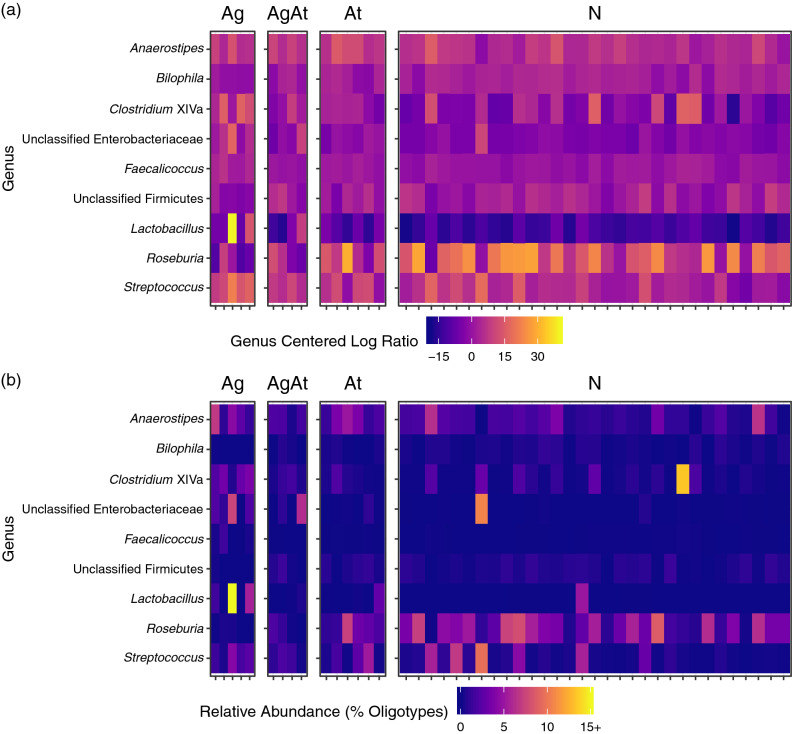
Differentially abundant genera identified among 46 participants receiving outpatient addiction treatment, 2016–2017. We used ALDEx2 to identify nine genera that were differentially abundant between participants who used opioid agonists (Ag) vs. participants who used neither agonists nor antagonists (N). Differentially abundant genera had false discovery rate (FDR) corrected *p* values < 0.05 for Wilcoxon rank sum tests comparing centered log ratios (**a**) computed from genera abundance (sample profiles are plotted as columns in heatmaps, rows represent the nine taxa). The corresponding relative abundance of each taxa is shown in (**b**). *Clostridium* cluster XIVa (FDR *p* value: 0.033), unclassified Enterobacteriaceae (FDR *p* value: 0.026), *Lactobacillus* (FDR *p* value: 0.031), *Faecalicoccus* (FDR *p* value: 0.037), *Anaerostipes* (FDR *p* value: 0.040), and *Streptococcus* (FDR *p* value: 0.045) abundances were higher in Ag vs. N participants while *Roseburia* (FDR *p* value: 0.043), unclassified Firmicutes (FDR *p* value: 0.031), and *Bilophila* (FDR *p* value: 0.037) were less abundant in Ag vs. N participants. We found no statistically significant differences between other opioid agonist/antagonist groups. *Ag* agonist only, A*gAt* agonist–antagonist, *At* antagonist only, *N* neither agonist nor antagonist.

We identified nine differentially abundant genera between Ag and N participants (Fig. 3). Unclassified Enterobacteriaceae (FDR *p* value: 0.026), *Lactobacillus* (FDR *p* value: 0.031), *Clostridium* cluster XIVa (FDR *p* value: 0.033), *Faecalicoccus* (FDR *p* value: 0.037), *Anaerostipes* (FDR *p* value: 0.040), and *Streptococcus* (FDR *p* value: 0.045) abundances were higher in Ag vs. N participants. Unclassified Firmicutes (FDR *p* value: 0.031), *Bilophila* (FDR *p* value: 0.037), and *Roseburia* (FDR *p* value: 0.043) were less abundant in Ag vs. N participants. We found no statistically significant differences between AgAt vs. N or At vs. N participants. In sensitivity analyses examining the second and third study samples, *Roseburia* remained less abundant in Ag vs. N participants, and Clostridium XIVa, *Bacteroides*, and *Faecalibacterium* remained more abundant in Ag vs. N participants in the second sample (Supplemental Table S1). There remained no statistically significant differences between AgAt vs. N or At vs. N participants in second and third samples.

### Fiber intake and alcohol

To explore whether differences in alcohol or fiber intake could explain the gut microbiota differences we observed between Ag and N participants, we examined opioid agonist–antagonist use (Table 1), alpha diversity (Supplementary Fig. S8), de novo enterotypes (Supplementary Fig. S9), and genera abundance by dietary fiber intake and alcohol use in the 30 days before the substance use survey. Dietary fiber and alcohol use were lower among Ag participants, but did not statistically differ (Table 1). Gut microbiota richness was positively and linearly associated with fiber intake (Pearson correlation: 0.35, *p* = 0.02, Supplementary Fig. S8). ALDEx2 did not identify any differentially abundant genera by fiber intake or alcohol use.

## Discussion

In our study of 46 individuals receiving addiction treatment, exposure to opioid agonists was associated with several variations in human gut microbiota diversity, enterotypes, and taxa. Participants who only used opioid agonists had lower alpha diversity and different bacterial community profiles than participants who used neither agonists nor antagonists. These results were consistent with those from a murine model^7^ and should be explored in larger studies that can examine both microbial composition and metabolic differences associated with opioid agonist exposure.

While consistent with a murine model, our findings differed from work by Xu et al*.*, who noted increased microbiota diversity among 45 Chinese men with any substance use disorder (58% used heroin) compared to 48 healthy controls^7,13^. Our conflicting results likely reflect differences in study design and the populations compared. We recruited a sample of participants who were all receiving addiction treatment to help control for lifestyle and dietary factors associated with being in recovery. Further, all participants in our study had the possibility of obtaining an opioid antagonist prescription.

Participants exposed to opioid agonists were more likely to have a low diversity *Bacteroides* enterotype^46^. No participants exposed to opioid agonists, including those with concurrent antagonist exposure, had the *Prevotella* enterotype, which was previously associated with a fiber-rich diet^46^. Accordingly, this association could reflect lower fiber consumption among participants only exposed to opioid agonists, or could reflect underlying processes common to low fiber diets and opioid agonist exposure, including slowed transit time, constipation, and reduced stool water content^9,50,51^. We were unable to disentangle the impacts of fiber and opioid agonist exposure; however, murine models suggest that the gut barrier compromise and bacterial translocation caused by morphine exposure is not induced by a low fiber diet^8^. Future studies will need to further differentiate the impacts of fiber and opioids on the human gut microbiota.

The differences in the abundance of two bacterial genera, *Roseburia* and *Bilophila*, by opioid agonist exposure are potentially concordant with murine models suggesting that opioid exposure may impact inflammation and bacterial metabolism^6–8^. *Roseburia*, a bacterial genus that produces butyrate, was decreased among participants only exposed to opioid agonists^52^. Butyrate generally benefits colon health by reducing inflammation, oxidative stress, and promoting gut barrier health^52^. *Roseburia* is considered part of the *Clostridium* XIVa cluster, a functionally related group of bacteria that includes many genera. Seemingly contrary then, is the association of increased *Clostridium* XIVa abundance with opioid agonist use. A search of the RDP, the phylogenetic reference assignment database used to assign sequences to genera, suggests that this association reflects nuances in sequence taxa classification^40^. The *Clostridium* XIVa genus in RDP includes only three *Clostridium* species of unknown butyrate production status and does not include any *Roseburia* species^40^.

Our study was unable to perform the metabolomic analysis required to confirm that the lower abundance of *Roseburia* species observed in participants exposed to opioid agonists related to lower levels of butyrate in the gut environment. However, recent studies using murine models highlight that probiotic administration^11^ and potentially butyrate^6^ are associated with decreased opioid antinociceptive tolerance and that supplementation with omega-3 polyunsaturated fatty acids attenuates opioid-seeking behaviors and anxiety symptoms during opioid withdrawal^19^. These findings underscore the potential importance of modulating the gut environment to mitigate overdose risk and support OUD recovery. Future, larger studies should investigate both the abundance and metabolic activities of key microbes to help design effective dietary interventions for individuals with OUD.

Opioid agonist exposure was also associated with decreased *Bilophila*, a genus that uses bile as a nutrient source^53^. These decreases may correlate with the reduced intestinal primary and secondary bile acid levels observed in morphine exposed mice, which were accompanied by gut microbiota changes, gut barrier disruption, and systemic inflammation, though the temporal sequence of changes has not yet been determined^8^. This should be studied further, using sample storage conditions that allow for a direct examination of bile acid levels in opioid-exposed participants.

The remaining bacteria that were differentially abundant in participants exposed to opioid agonists may have been overly influenced by outliers and/or their functional profiles are less well characterized. Given current research in the area of psychopathology and the gut microbiota, *Lactobacillus* and *Bifidobacterium,* though *Bifidobacterium* was not included among the differentially abundant genera in the present analysis, may warrant further study. Opioid-related alterations in these genera were found to be reversible by omega-3 polyunsaturated fatty acid^19^ and probiotic administration^11^, and they exhibited bile-salt hydrolase activity^54^ in prior work. Moreover, certain *Lactobacillus* and *Bifidobacterium* species may impact anxiety and depressive symptoms^55–57^.

We did not identify any differences in microbiota diversity and taxa between participants with combined agonist and antagonist (AgAt) exposure or antagonist only exposure (At) and participants with neither agonist nor antagonist exposure (N). These results align with the observed antagonism of morphine’s effects by naltrexone in mice^7^. Together, these results suggest that the combinations of agonists and antagonists used clinically may have benefits beyond their intended applications (e.g., naloxone reducing the abuse potential and risk of overdose for buprenorphine). These benefits to gut health may overlap with the other clinical uses of naltrexone, including relief from opioid-induced constipation, the potential promotion of gut mucosal healing for Crohn’s disease, and anti-inflammatory applications for treating chronic pain^21–25^. However, the mechanisms of naltrexone’s action in Crohn’s disease are the subject of some debate, and the dose of naltrexone used in these applications is lower than the dose recommended for craving management in alcohol and OUDs^23,25^. Nonetheless, these applications suggest the possibility that naltrexone improves gut barrier function, which is increasingly explored as a determinant of psychopathology and recovery^3–5^. Further, it is unknown whether naltrexone’s effectiveness in mitigating cravings to use alcohol or opioids in the context of addiction treatment is partially explained or supported by these potential benefits to gut barrier function.

The low bioavailability of naloxone in buprenorphine–naloxone formulations taken orally^27^ highlights the importance of considering other reasons, beyond naloxone’s antagonizing activity, to explain the lack of differences between the AgAt and N groups. This finding could reflect that buprenorphine, the most common agonist exposure in our study’s AgAt group, is a partial opioid agonist^26^, whereas much of the currently available research focuses on full agonists, such as morphine^7,11^, heroin^13^, and many prescription opioids (though tramadol is a notable exception)^12^. Future research should examine how opioid potency and dosing impact the gut microbiota.

This study was limited by the small sample size (n = 46) and small number of agonist and antagonist exposed participants. Further, we did not include healthy controls, but the bacterial communities of 44 of 46 samples were consistent with bacterial communities observed from two large studies of healthy humans, HMP and MetaHIT. We were not able to adjust for confounding given our small sample size, and instead presented descriptive statistics highlighting the potential extent of confounding by fiber and alcohol use. Despite this, we found several biologically plausible associations that merit further exploration in larger studies, particularly with more participants using opioids.

Many of the genera that were differentially abundant by opioid exposure group were either less well described in the literature or may have been overly influenced by outliers, complicating interpretation of observed associations. In particular, one participant had extremely high *Lactobacillus* levels that may have been related to probiotic use, which we unfortunately did not measure. This limited our ability to examine the association of *Lactobacillus* abundance with opioid exposure and further, to hypothesize about how the bile salt hydrolase activity of this genera may relate to the existing literature on levels of primary and secondary bile salts following opioid exposure^8^.

Nearly all measures were self-reported and are therefore subject to recall bias. Several participants were lost to follow-up, which opens the potential for selection bias if participants who dropped out had more severe substance use disorders or otherwise systematically differed from retained participants. Participants who used opioid agonists likely had harder stool and slowed transit time, but it is unknown if this affected our results.

Our study had several offsetting strengths. We used analytic tools appropriate for the compositional nature of microbiota data to avoid bias^58^. This is the first study to describe enterotypes among humans with OUDs and we included reference-based enterotypes that allow comparison of our results to those from other disorders to identify common mechanisms underlying the dysbioses we detected. We evaluated the impact of two factors that could alternatively explain the associations observed between the gut microbiota and opioids: alcohol and dietary fiber. Neither exhibited as strong a relationship as the results for opioids. Finally, our findings around the microbiota features associated with opioid use were largely consistent in sensitivity analyses that examined stool samples collected up to three weeks after the 46 samples included in our main analyses.

## Conclusions

In conclusion, individuals exposed to opioid agonists had differences in gut microbiota diversity, enterotypes, and bacterial genera compared to individuals who used neither an agonist nor antagonist. We observed decreased diversity and richness, an absence of the *Prevotella* enterotype, and decreased *Roseburia,* a butyrate producer, and *Bilophila*, which could relate to the bile acid dysregulation observed in murine models^8,9^. Individuals who concurrently used an opioid agonist and antagonist, and individuals who only used an opioid antagonist did not differ in gut microbiota diversity, richness, or genera relative abundance compared to individuals who used neither an agonist nor antagonist. These findings suggest that the effects of opioids on the gut microbiota might be antagonized by naltrexone or naloxone or that partial opioid agonists may differentially impact the microbiota than full agonists. Further characterization of the relationships between opioid agonist and antagonist exposure, gut permeability, inflammation, and relapse predictors could inform whether psycho-adjunctive treatments for OUDs might improve addiction treatment outcomes and modify antinociceptive tolerance to opioids.

## Supplementary information

Supplementary Information 1.

## Data Availability

The datasets generated during and/or analysed during the current study are available on reasonable request to the corresponding author. The supplementary material provides details about the study’s methods and results.
