# Supplementary material for: Opioid agonist and antagonist use and the gut microbiota: associations among people in addiction treatment
Source: Sci Rep. 2020 Nov 10;10:19471. doi: 10.1038/s41598-020-76570-9 (PMC7655955; doi:10.1038/s41598-020-76570-9)
Supplement: Supplementary file 1 — Supplementary Information 1. [file 41598_2020_76570_MOESM1_ESM.pdf]

Opioid Agonist and Antagonist Use and the Gut Microbiota: Associations among People in Addiction Treatment

Scientific Reports

Rachel E. Gicquelais[1-4]*, Amy S.B. Bohnert[4-6], Laura Thomas[4,5] and Betsy Foxman[7]

[1]University of Wisconsin-Madison School of Nursing, 701 Highland Avenue, Madison, WI 53705, gicquelais@wisc.edu
[2]Department of Epidemiology, Johns Hopkins Bloomberg School of Public Health, 615 N. Wolfe St, Baltimore, MD, 21205
[3]Department of Epidemiology, University of Michigan School of Public Health, 1415 Washington Heights, Ann Arbor, MI 48109
[4]Department of Psychiatry, University of Michigan Medical School, 2800 Plymouth Rd, Ann Arbor, MI, 48109
[5]VA Center for Clinical Management Research, 2800 Plymouth Rd, Ann Arbor, MI, 48109
[6]Department of Anesthesiology, University of Michigan Medical School,
[7]Department of Epidemiology, University of Michigan School of Public Health, 1415 Washington Heights, Ann Arbor, MI 48109, bfoxman@umich.edu

Supplementary Information

Supplementary Methods 3
Methods Overview 3
Participant Recruitment 3
Substance Use Assessment for Microbiota Study Eligibility 3
Measures: Participant Characteristics 4
Opioid Agonist and Antagonist Use 4
Depression, Anxiety, and Cravings to Use 5
Microbiota Study Population Characteristics 5
Dietary Fiber Intake Estimation 5
Stool Sample Collection and Sequencing 6
Stool Sample Collection 6

DNA Extraction and Illumina MiSeq Sequencing 7

Microbiota Measures and Analyses 9

Genus Relative Abundance and Diversity Metrics 9

De Novo and Reference-Based Enterotyping 10

Genus Differential Abundance by Opioid Agonist and Antagonist Use 12

Supplementary Fig. S1 Inclusion Criteria and Opioid Use among 46 Participants Enrolled from an Outpatient Addiction Treatment Facility into a Study of the Gut Microbiota and Opioid Use, 2016-2017 13

Supplementary Fig. S2 Alpha Diversity was Relatively Stable Over a One Month Follow-Up Period (Across up to 3 Sample Time Points) for Most Participants Enrolled from an Outpatient Addiction Treatment Facility, 2016-2017 14

Supplementary Fig. S3 Gut Microbiota Beta Diversity among 46 Participants Enrolled from an Outpatient Addiction Treatment Facility by Opioid Agonist and Antagonist Use, 2016-2017 15

Supplementary Fig. S4 Beta Diversity during Up to Three Study Visits Demonstrated Clustering By Participant 16

Supplementary Fig. S5 Dirichlet Multinomial Mixture Model Fit (Laplace Approximation) for Models with 1-5 Enterotypes 17

Supplementary Fig. S6a Enterotypes Recovered from *De Novo* Clustering of Gut Microbiota Samples among 46 Participants Enrolled from an Outpatient Addiction Treatment Facility, 2016-2017 18

Supplementary Fig. S6b Heatmap of Top 20 Oligotypes that Differentiated Three Enterotypes Recovered from *De Novo* Clustering of Gut Microbiota Samples among 46 Participants Enrolled from an Outpatient Addiction Treatment Facility, 2016-2017 19

Supplementary Fig. S7 Alpha Diversity in *De Novo* Assigned Gut Microbiota Enterotypes among 46 Participants Enrolled from an Outpatient Addiction Treatment Facility by Opioid Agonist and Antagonist Use, 2016-2017 20

Supplementary Fig. S8 Gut Microbiota Alpha Diversity by Alcohol Use and Fiber Intake among 46 Participants Enrolled from an Outpatient Addiction Treatment Facility, 2016-2017 21

Supplementary Fig. S9 *De Novo* Assigned Enterotypes by Alcohol Use and Dietary Fiber Intake among 46 Participants Enrolled from an Outpatient Addiction Treatment Facility, 2016-2017 22

Supplementary Table S1 Comparison of Results for Association of Agonist-Antagonist Exposure with Microbiota Features Across Samples Submitted Across 3 Study Visits from 46 Participants Enrolled from an Outpatient Addiction Treatment Facility, 2016-2017 23

References 26

Supplementary Methods

Methods Overview

We consented 46 patients aged 18-60 years who were receiving treatment in an outpatient addiction treatment facility to participate in a gut microbiota and substance use study (Supplementary Fig. S1). We surveyed participants about the substances they used in the 30 days prior and reviewed participant medical records to characterize their opioid agonist and antagonist use. Participants submitted a stool sample and we sequenced the V4 hypervariable region of the 16S ribosomal ribonucleic acid (rRNA) gene to characterize the gut microbiota. Each participant's opioid agonist and antagonist use patterns from survey and medical record data were summarized as agonist only (Ag), combined agonist-antagonist (AgAt), antagonist only (At), or neither agonist nor antagonist (N). We compared gut microbiota diversity, enterotypes, and genera relative abundance between participants in these four opioid agonist and antagonist groups. The study was approved by the Institutional Review Board at the University of Michigan (HUM00113964).

Participant Recruitment

Substance Use Assessment for Microbiota Study Eligibility

We used an abbreviated version of the Alcohol, Smoking, and Substance Involvement Screening Test (ASSIST) to assess past 30-day substance use. ASSIST is a 12-item questionnaire that asks about use of street opioids, opioid pain medications (with or without a prescription), methadone or buprenorphine-naloxone (with or without a prescription), tobacco, alcohol, cannabis, cocaine (including crack), amphetamines, inhalants, sedatives or sleeping pills, and hallucinogens[1,2]. Participants who only endorsed tobacco were not eligible for the microbiota study without concurrent reported use of another substance. Participants reporting past 30-day

use (nonmedical or as prescribed) of medications for opioid use disorder (buprenorphine-naloxone or methadone) were eligible, regardless of past 30-day use of other substances. We used a modified version of the Current Opioid Misuse Measure (COMM) to capture self-reported misuse of prescribed opioids[3]. The COMM is an eight-item scale that assesses self-reported opioid misuse not captured by ASSIST[3]. Participants with a score >0 for questions 4 and 6-8 were eligible based on self-reported prescription opioid misuse regardless of their past 30-day substance use from the ASSIST[3].

Measures: Participant Characteristics

Opioid Agonist and Antagonist Use

We used a combination of the ASSIST and medical record review to identify any opioid agonist use. We defined opioid agonist use as a binary indicator for either of the following: 1) self-reported opioid use (heroin, methadone, buprenorphine, or prescription opioids used as prescribed or not as prescribed) in the 30 days before study enrollment on the modified ASSIST, or 2) buprenorphine-naloxone use during the day of sample collection in the medical record. We supplemented the ASSIST data on buprenorphine use with data from the medical record because survey questions about medications for opioid use disorder were added to the modified ASSIST during January 2017 and were not available prior. Of note, one participant who was prescribed opioids to manage pain was included in the group of participants who used opioid agonists.

We also assessed opioid antagonist use. For the purpose of this study, we defined opioid antagonist use as either of the following according to the medical record: 1) buprenorphine-naloxone use during the day of sample collection, or 2) naltrexone use during the day of sample collection. We included any formulation of either opioid antagonist. Finally, we created a

categorical variable describing the overlap in opioid agonist and antagonist use (i.e., agonist only, combined agonist-antagonist, antagonist only, and neither agonist nor antagonist).

Depression, Anxiety, and Cravings to Use

We summarized scores from the Patient Health Questionnaire-9 (PHQ-9), a reliable and valid depression severity screening tool (Cronbach's α: 0.86-0.89, Test-retest correlation: 0.84, range: 0-27)[4]. We also summarized scores from the Generalized Anxiety Disorder 7-Item Scale (Cronbach's α: 0.92, Test-retest correlation: 0.83, range: 0-21)[5]. For reference, a sum $\geq$10 indicated possible GAD[5]. Finally, cravings to use drugs or alcohol were reported using summed scores from a modified version of the Penn Alcohol Craving Scale adapted to capture cravings to use drugs (range: 0-30)[6–8].

Microbiota Study Population Characteristics

We summarized average age, self-identified gender (female, male, and other), race (black, white, other, or multiple races), ethnicity (Hispanic vs. non-Hispanic), and self-reported days in SUD treatment at the study site. We used the ASSIST to describe alcohol use during the 30 days before completing the substance use survey[1]. Finally, we summarized self-reported antibiotic use during the week of sample collection.

Dietary Fiber Intake Estimation

Dietary fiber intake has previously been associated with the gut microbiota, transit time, and stool water content[9–12]. We therefore calculated predicted past-month dietary fiber intake (grams per day) from food frequency questions completed at enrollment in the microbiota study using validated predictive models developed from the National Health and Nutrition

Examination Survey (NHANES)[13]. Upon enrollment in the microbiota study, participants completed a validated food frequency questionnaire about general past month intake of 25 food and drink items[14]. Due to a skipped question on the survey, participants did not report their cheese intake. We therefore assigned participants the survey weighted mean values of cheese intake using age and race-specific values from the 2009-2010 cycle of the NHANES, which included the food frequency questionnaire used in our study. We did not use sex-specific values as cheese intake did not differ by sex in NHANES. We calculated predicted past month dietary fiber intake (predicted grams per day) from reported dietary item intake and validated predictive models developed from the National Health and Nutrition Examination Survey[13].

Stool Sample Collection and Sequencing

Stool Sample Collection

We based our stool collection protocols on those from Feigelson *et al.*, Flores *et al.*, and Fu *et al.*[15–17]. Participants self-collected stool samples by placing two dime-sized scoops of stool into a sterile Sarstedt tube with a spoon lid (Sarstedt, Nümbrecht, Germany) containing a cryopreservant, RNA*later*™ (Ambion, Austin, TX), and 5-10 glass beads (Walter Stern, Washington, NY). Participants then secured the lid, homogenized the sample in RNA*later*™ by shaking, and stored the sample at room temperature for up to two days before returning the sample to a research assistant. Previous research supported that RNA*later*™ preserved the composition of the stool bacterial community at room temperature for up to three days after collection[16,17]. Stool samples were then frozen in 1 mL aliquots at -80C.

DNA Extraction and Illumina MiSeq Sequencing

After all participants were enrolled, we thawed a 1 mL aliquot of each stool sample, centrifuged at 10,000xg, and resuspended in 1 mL 1X phosphate buffered saline (Gibco, Thermo Fisher Scientific, Waltham, MA, pH 7.4). We added 250 uL resuspended stool to each of two wells of bead beating plates and provided samples to the Microbial Systems Molecular Biology Laboratory at the University of Michigan. They used standard protocols for deoxyribonucleic acid (DNA) extraction and Illumina MiSeq sequencing of the V4 hypervariable region of the bacterial 16S ribosomal ribonucleic acid (rRNA) gene[18–20].

First, DNA was extracted using the Qiagen MagAttract PowerMicrobiome kit (Qiagen, Hilden, Germany). DNA libraries for the V4 region of the 16S rRNA gene were generated and 16S DNA was amplified using polymerase chain reaction (PCR) and barcoded dual-index primers for the V4 hypervariable region[20]. Reactions included 5 uL of 4 uM equimolar primer set, 0.15 uL AccuPrime *Taq* DNA High Fidelity Polymerase, 2 uL 10X AccuPrime PCR Buffer II (Thermo Fisher Scientific), 11.85 uL PCR-grade water, and 1 uL DNA and used the following cycling conditions: 2 min at 95°C, 30 cycles of 95°C for 20 s, 55°C for 15 s, and 72°C for 5 min, and finally 72°C for 10 min. PCR reactions were normalized, pooled, and quantified. The pooled amplicon library was sequenced using Illumina MiSeq with the 500 cycle MiSeq V2 Reagent kit (Illumina, San Diego, CA) with modifications to the primer set (custom read 1/read 2 and index primers were added to the reagent cartridge). Two types of mock communities were included with samples to assess sequencing error rates. Mocks included a community of 10 species (added as bacterial DNA, Zymo Research, Irvine CA, catalog no. D6300) and a mock community of two species added as suspended overnight cell culture in brain heart infusion broth (equal ratios of

Escherichia coli and *Staphylococcus aureus*). All samples were sequenced in duplicate and mocks were sequenced on each of four DNA extraction plates.

We processed sequencing reads using mothur (v1.39.5) and the MiSeq standard operating procedure (https://www.mothur.org/wiki/MiSeq_SOP, accessed November 8, 2017) to perform quality filtering and align sequences to the V4 region of the 16S rRNA gene[20]. We converted sequences to the format required for oligotyping and clustered samples into oligotypes using the procedures and default parameters described by Eren *et al.*[21,22]. Oligotyping uses minimum entropy decomposition methods to identify highly variable nucleotide positions and clusters sequences based on Shannon entropy[21]. Compared with operational taxonomic units, an alternative way to cluster sequences based on distance-based metrics, oligotyping improves identification of taxa at the species or strain level[21].

Two samples with fewer than 1,000 reads were removed from further analysis; however, we were able to retain these two participants for analysis using their duplicate sequenced sample. We verified that all mock communities resembled their known compositions (data not shown) and summed duplicate sequenced samples. For this analysis, we examined the first sample submitted per participant, amounting to 46 samples with 2,207,827 sequence reads (21,796 – 77,013 reads per participant) and 354 oligotypes. The full analytic dataset, which was used in sensitivity analyses comparing results from the first collected sample to those collected at study visits 2 and 3, comprised 129 samples (total reads: 5,745,902, range: 7,043 – 87,403 per participant). We assigned oligotype taxonomy using the Ribosomal Database Project (RDP, release 11, update 5)[23]. We chose to focus main analyses on the samples from the first study visit per participant as this time point represented the most proximal stool microbiota data to the

ASSIST substance use inventory. Sensitivity analyses examined whether data from second and third study visits corroborated main findings from analyses of the first submitted study sample.

Microbiota Measures and Analyses

Genus Relative Abundance and Diversity Metrics

We calculated the relative abundance of genera in each sample (i.e., the number of sequencing reads from each genus divided by the total number of sequencing reads per sample). For alpha diversity analyses, we normalized sequencing depth across samples using rarefaction[24,25]. Briefly, the oligotype table was sampled without replacement to 90% of the minimum sequencing depth across all 129 samples sequenced as part of the study (i.e., sampled to a sequencing depth of 6,338 reads). Rarefaction was repeated 100 times, and alpha diversity metrics were averaged across the 100 replicates. Because the application of rarefaction in microbiome research is the subject of some debate[26], we also analyzed alpha diversity metrics on the non-rarefied oligotype table as a sensitivity analysis. Alpha diversity metrics included Shannon diversity and the Chao1 Index, which summarized within-sample oligotype diversity (number and evenness of oligotypes) and richness (number of oligotypes), respectively. Further, we visualized between-sample (beta diversity) differences using principal component analysis of the Aitchison Distance metric, which visualizes Euclidean distance using a centered log ratio transformation of the oligotype table, an approach consistent with the compositional nature of the data (described further below)[27].

Microbiota sequencing data are compositional; the total number of sequences from each sample is bound by an arbitrary depth of sequencing coverage that does not reflect the true abundance of bacteria in the participant's gut[28]. Thus, taxa comparisons between samples must

be analyzed on the relative (not absolute) scale[28]. Failure to analyze these data as compositional results in spurious correlations between taxa and biases associations of taxa abundance with other taxa and covariates[28]. We therefore applied three analytic approaches to compare taxa distributions by covariates of interest that accounted for the compositional nature of the data: the Aitchison Distance described above, and Dirichlet multinomial mixture modeling and ALDEx2, described further below[29–31].

De Novo and Reference-Based Enterotyping

Enterotyping distills highly dimensional microbiota taxa data into clusters (i.e., groups of samples exhibiting similar taxa distributions) by leveraging the information from the covariance structure of taxa[9,30,32]. The high dimensionality of microbiome data (number of taxa) poses a challenge during analysis, as testing for associations of single taxa with metadata requires many tests and results can be difficult to interpret without acknowledging co-occurring changes in other taxa. Enterotyping addresses these challenges through data reduction and summary by clustering samples with similar bacterial profiles into groups based on the distribution of taxa in samples[9].

We applied two clustering approaches that classified each stool sample's genus-level read counts into enterotypes[9,30]. First, we used Dirichlet multinomial mixture (DMM) models, an extension of latent profile analysis adapted for microbiota data by Holmes *et al.*[30]. Like traditional latent profile analysis, this technique recovers unobserved (i.e., latent) subgroups based on joint distributions of bacterial genera. DMM-based enterotypes were assigned *de novo* (i.e., based on the data and participants included in our study) using data from all 129 samples collected during the study to standardize DMM assignments across all samples. We compared

model fit using the Laplace approximation of negative log models for DMM models with one to five enterotypes and chose the number of enterotypes that optimized model fit (i.e., minimized the Laplace approximation)[30]. We then assigned each sample to its most likely enterotype based on posterior probabilities of enterotype assignment. All 46 samples used in the main analysis had posterior probabilities ≥90% and were therefore all assigned to an enterotype (minimum posterior probability: 95.3%). We examined the average relative abundance of all genera and the top 20 taxa that were most influential in distinguishing between enterotypes to summarize taxa distributions representative of each enterotype.

Costea *et al*. recently reviewed the enterotyping literature and suggested the existence of three enterotypes in healthy human populations based on their dominant bacterial taxa: *Bacteroides*, Firmicutes, and *Prevotella* and created an online reference-based enterotyping tool that assigns uploaded samples to one of these three enterotypes[9].This tool compares genera relative abundance in uploaded samples to relative abundance from two studies of the healthy human gut microbiota, the Human Microbiome Project (HMP) and the Metagenomics of the Human Intestinal Tract (MetaHIT) study. It also applies enterotyping methods described by Arumugam *et al*. (i.e., partitioning around medoid clustering) to assign an enterotype based those observed in HMP and MetaHIT[9,32].

We uploaded genus relative abundance data from our study to http://enterotypes.org and obtained enterotype assignments and a binary variable indicating whether each sample had similar genera to the observed patterns in reference samples from HMP and MetaHIT. Two of 46 samples in the main analysis were not comparable to reference samples based on this indicator and were therefore assigned as “missing” for their assignment.

Genus Differential Abundance by Opioid Agonist and Antagonist Use

We compared abundance of specific genera by opioid use status and other covariates of interest using ALDEx2, an analysis of variance-like tool for compositional data[31]. For each genus, ALDEx2 inferred absolute abundance given the observed abundance matrix over 1,000 Monte-Carlo simulations from a Dirichlet distribution. To account for the compositional structure of the data, genera abundances for each sample and simulation were transformed to centered log ratios (i.e., log of the ratio of $taxa_i$ abundance for sample *j* divided by the geometric mean for the abundance of all taxa [i=1:K] in sample *j*). ALDEx2 then calculated each genus' median centered log ratio by group (e.g., opioid agonist use vs. no use) for each simulation. Within-group variability in each taxa's log ratios reflected sampling variation whereas between-group differences represented the biological variation of interest (e.g., by opioid agonist-antagonist use status). These were used to calculate an effect size for each genus as the median difference in centered log ratios between groups across all simulations divided by the median of the largest detected difference within groups for each condition (e.g., the median of two items: 1) the largest difference in centered log ratios for $taxa_i$ among opioid agonist use group across 1,000 simulations and 2) the largest difference in centered log ratios for $taxa_i$ among people not using opioid agonists across 1,000 simulations). Statistical significance for each effect size was summarized as a p-value corrected for the false discovery rate (FDR) using the Benjamini-Hochberg procedure. We summarized genera centered log ratios and relative abundance for all samples as heat maps (Fig. 3) for taxa with FDR corrected p-values<0.05 identified from the ALDEx2 Wilcoxon rank sum test.

Supplementary Fig. S1 Inclusion Criteria and Opioid Use among 46 Participants Enrolled from an Outpatient Addiction Treatment Facility into a Study of the Gut Microbiota and Opioid Use, 2016-2017

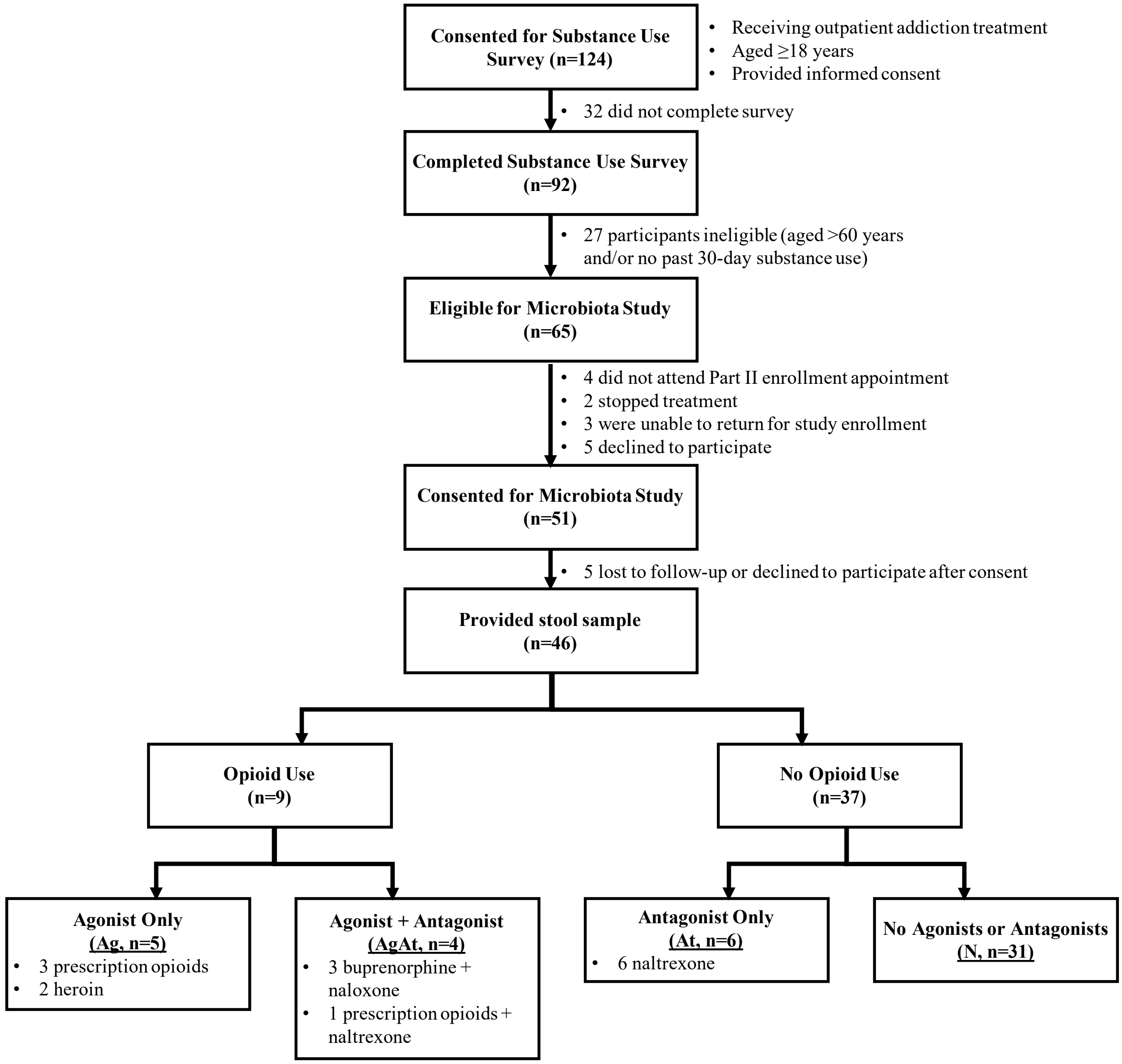

We included 46 participants who were eligible and provided informed consent for both study stages and provided stool samples in the present analysis. Among the 9 using opioids, 5 used only agonists (prescription opioids or heroin) and 4 used an agonist-antagonist combination during the time their sample was provided. Among the 37 not using opioids, 6 used an opioid antagonist (naltrexone) and 31 were not exposed to neither opioid agonists nor antagonists during the time they provided their stool sample.

Supplementary Fig. S2 Alpha Diversity was Relatively Stable Over a One Month Follow-Up Period (Across up to 3 Sample Time Points) for Most Participants Enrolled from an Outpatient Addiction Treatment Facility, 2016-2017

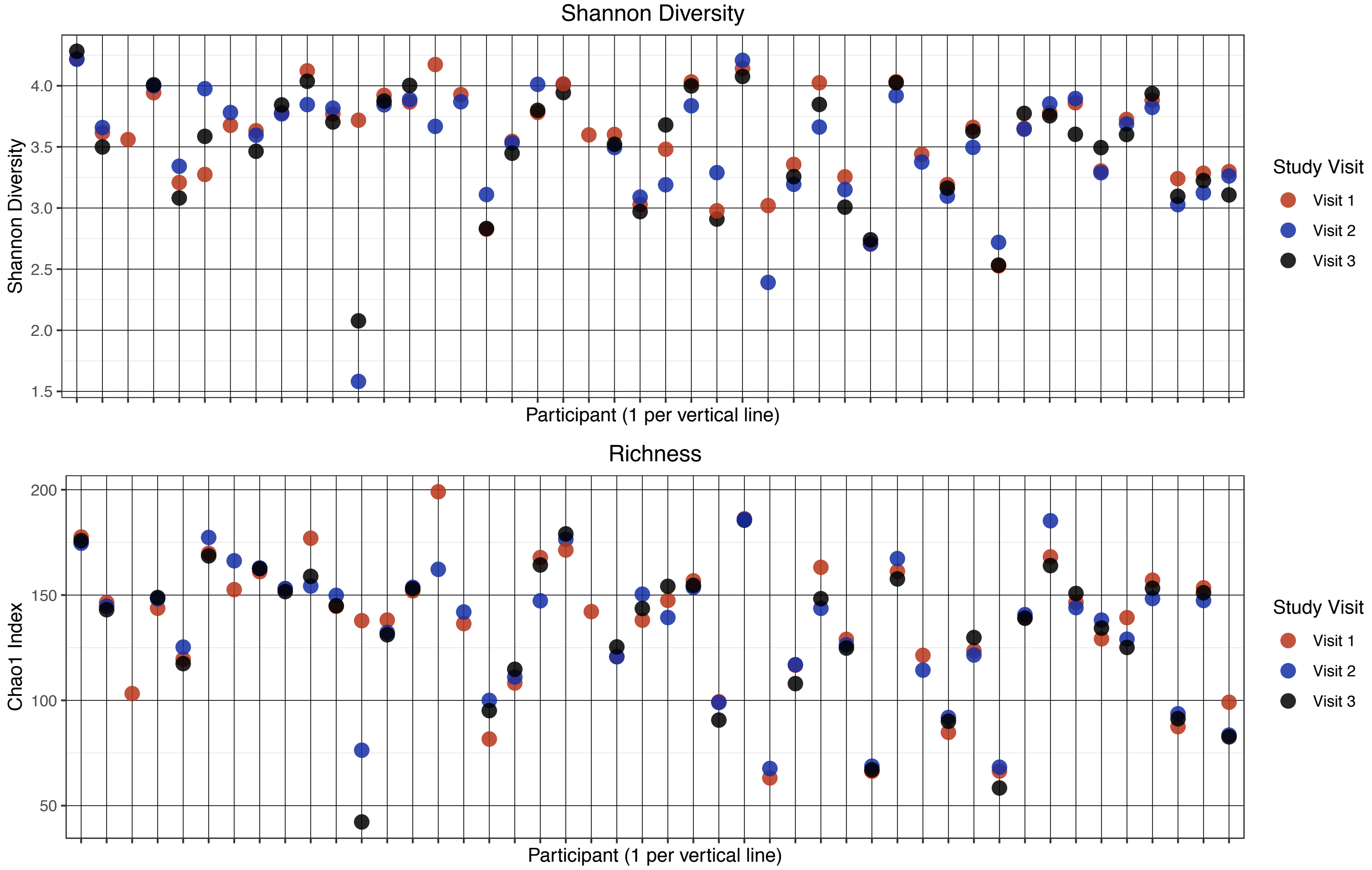

Participants submitted up to three samples during a month-long study period. The plot shows participant-level alpha diversity metrics (1 participant per x-axis tick mark and plot-area vertical line, with points indicating study visit alpha diversity metrics on the y-axis). We found little difference in alpha diversity (Shannon Diversity [top] and richness [bottom, Chao1]) over time for most participants.

Supplementary Fig. S3 Gut Microbiota Beta Diversity among 46 Participants Enrolled from an Outpatient Addiction Treatment Facility by Opioid Agonist and Antagonist Use, 2016-2017

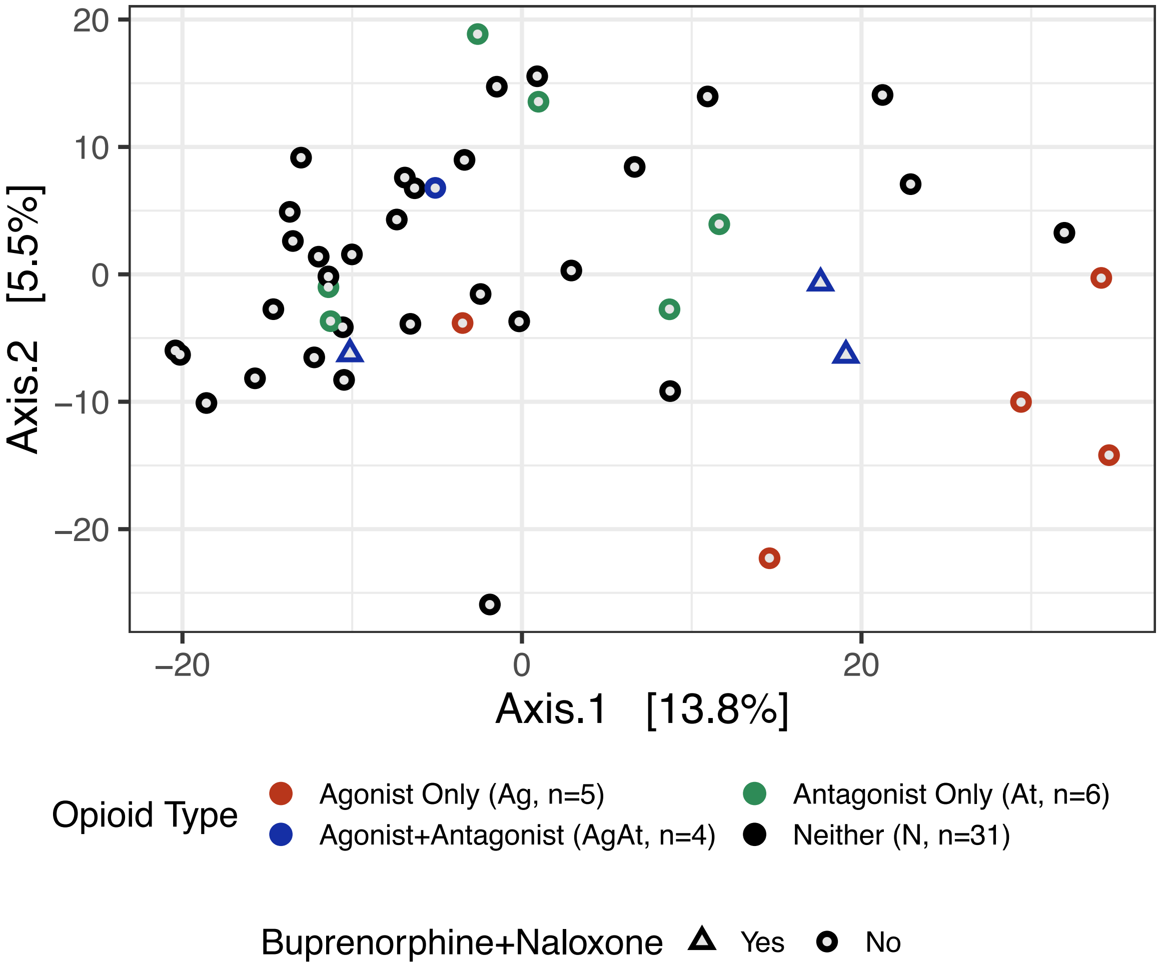

We summarized beta diversity using Aitchison Distance and a principal component analysis. Strong clustering was not observed by opioid agonist-antagonist groups. For this reason, we avoided further statistical testing.

Supplementary Fig. S4 Beta Diversity during Up to Three Study Visits Demonstrated Clustering By Participant

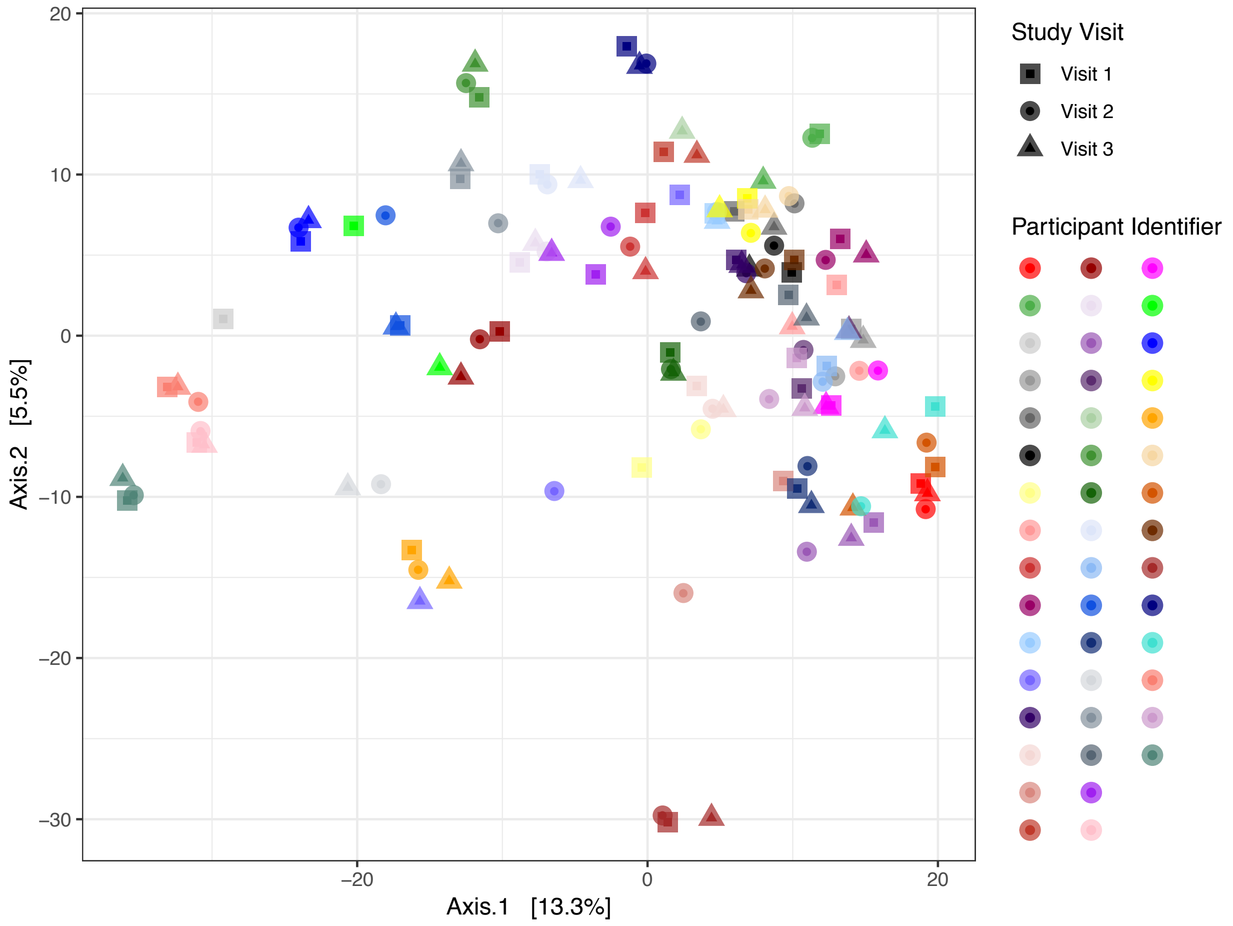

Participants submitted up to three samples during the month-long microbiota study period. Samples from different time points clustered strongly by participant using the Aitchison distance metric and principal component analysis.

Supplementary Fig. S5 Dirichlet Multinomial Mixture Model Fit (Laplace Approximation) for Models with 1-5 Enterotypes

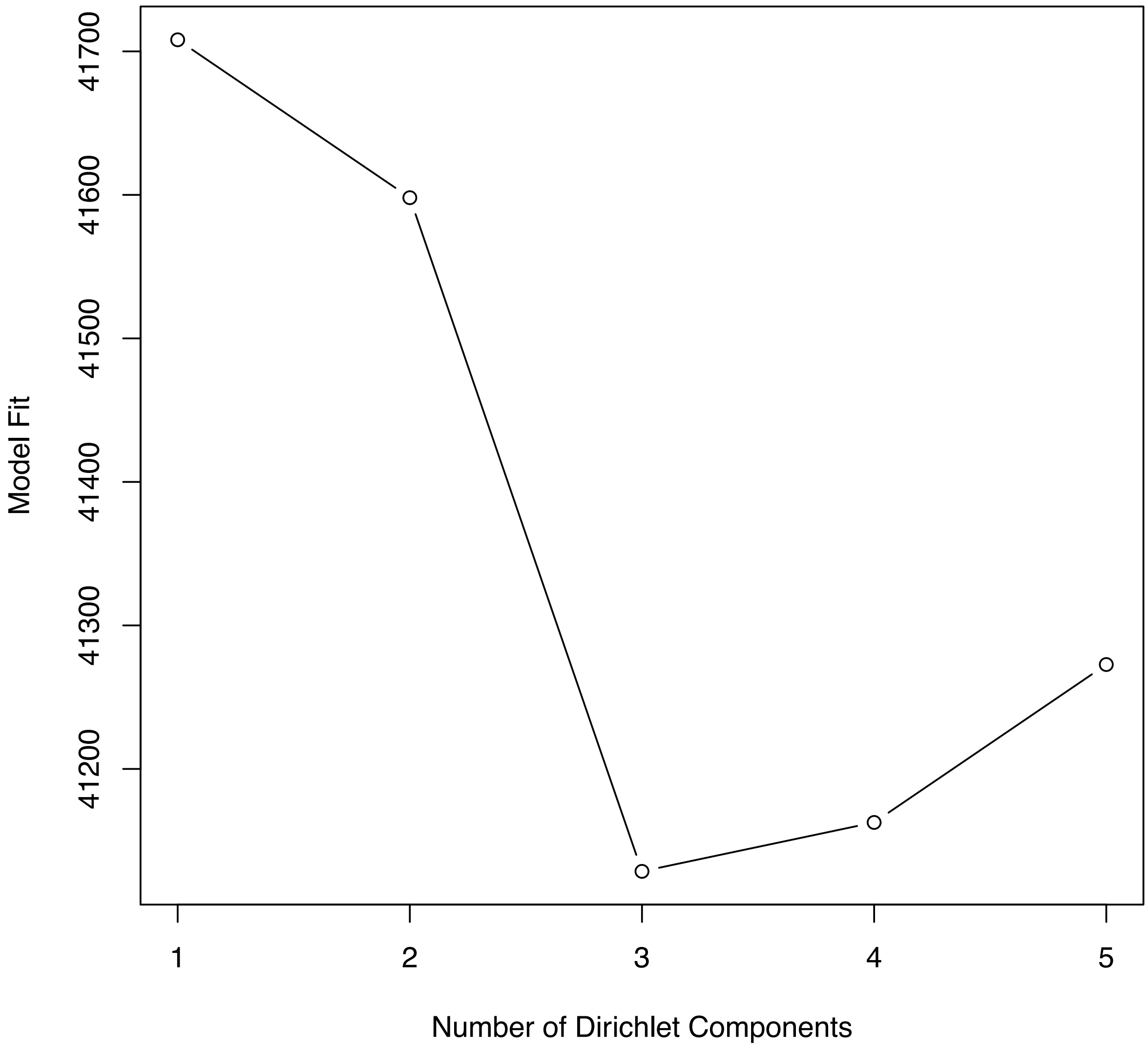

We fit Dirichlet multinomial mixture models with one to five enterotypes. Model fit was optimized by a three enterotype model, which minimized the Laplace approximation.

Supplementary Fig. S6a Enterotypes Recovered from *De Novo* Clustering of Gut Microbiota Samples among 46 Participants Enrolled from an Outpatient Addiction Treatment Facility, 2016-2017

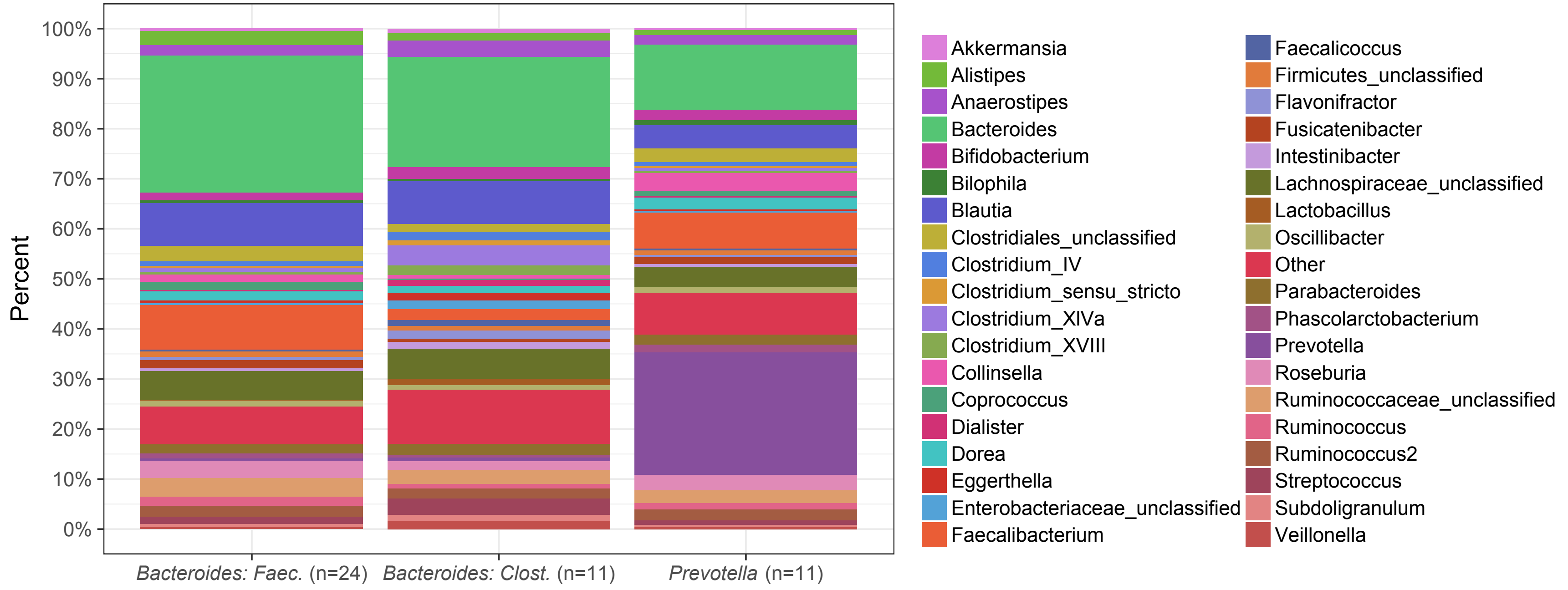

We identified two *Bacteroides* enterotypes, one with increased *Faecalibacterium* and a second with increased *Clostridium* cluster XIVa. The third enterotype was dominated by *Prevotella.* As such, *Prevotella* and *Bacteroides* were the first and second-most influential genera in assigning enterotypes, respecitively. Twenty-four participants assigned to the *Bacteroides: Faec.* enterotype had higher *Faecalibacterium* (mean *Faecalibacterium* relative abundance of 8.9% in *Bacteroides: Faec.*, 2.1% in *Bacteroides: Clost.*, 7.2% in *Prevotella*). *Faecalibacterium* was the third most influential genera in assigning enterotypes. Eleven participants assigned to the *Bacteroides: Clost.* enterotype had higher *Clostridium* cluster XIVa (mean *Clostridium* cluster XIVa relative abundance of 4.0% in *Bacteroides: Clost.*, 0.86% in *Bacteroides: Faec.*, and 0.63% in *Prevotella* enterotype). *Clostridium* cluster XIVa was the fifth most influential genera in assigning enterotypes. The fourth most influential genera in assigning enterotypes was *Blautia*, which distinguished the *Prevotella* and two *Bacteroides* enterotypes (mean *Blautia* relative abundance of 8.5% in *Bacteroides: Faec.*, 8.6% in *Bacteroides: Clost*, and 4.7% in *Prevotella*).

Supplementary Fig. S6b Heatmap of Top 20 Oligotypes that Differentiated Three Enterotypes Recovered from *De Novo* Clustering of Gut Microbiota Samples among 46 Participants Enrolled from an Outpatient Addiction Treatment Facility, 2016-2017

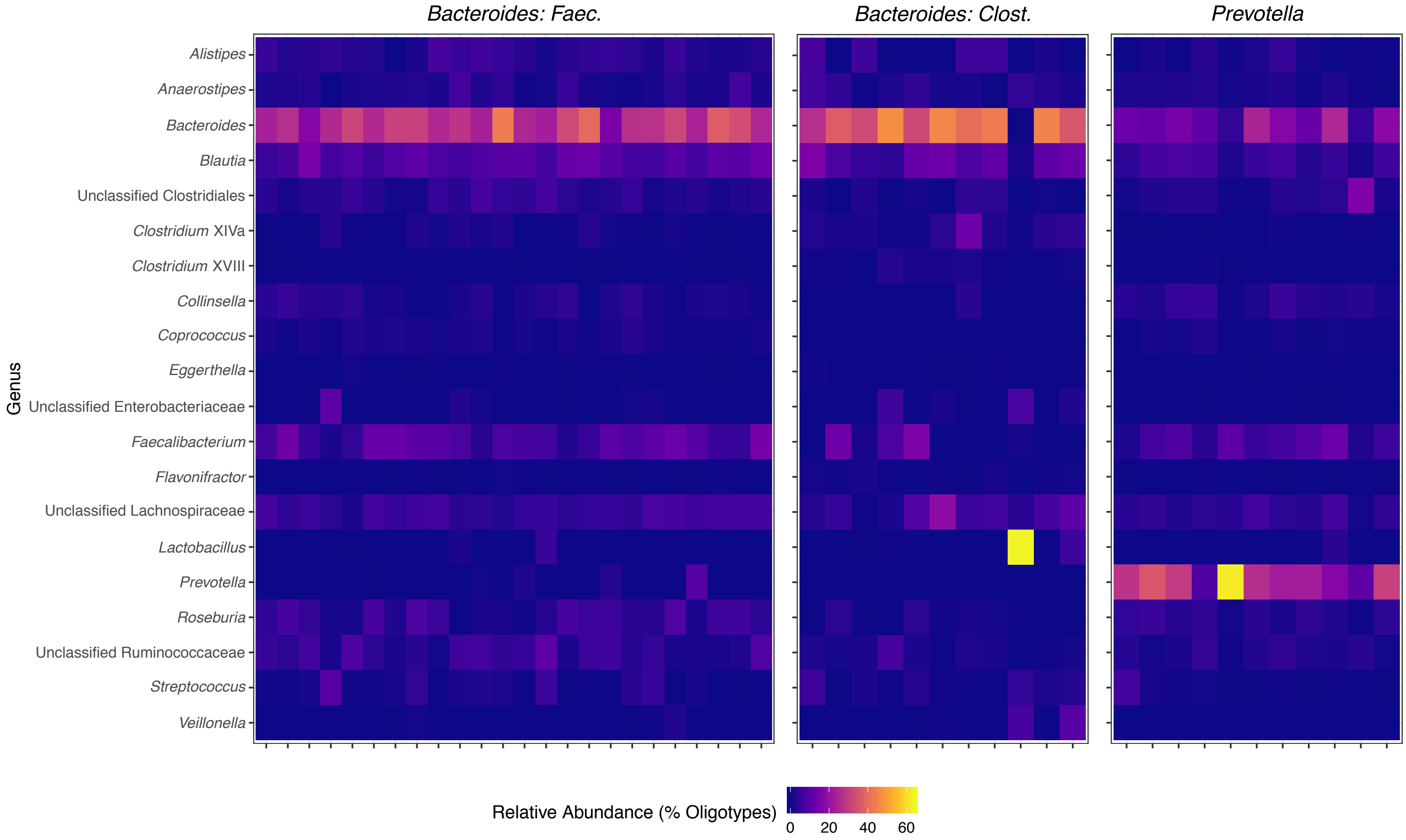

Heatmap of the relative abundance of the top 20 oligotypes that determined assignment of *de novo* enterotypes using Dirichlet Multinomial Mixture modeling. Plotted as participant samples on the x-axis (1 column per participant).

Supplementary Fig. S7 Alpha Diversity in *De Novo* Assigned Gut Microbiota Enterotypes among 46 Participants Enrolled from an Outpatient Addiction Treatment Facility by Opioid Agonist and Antagonist Use, 2016-2017

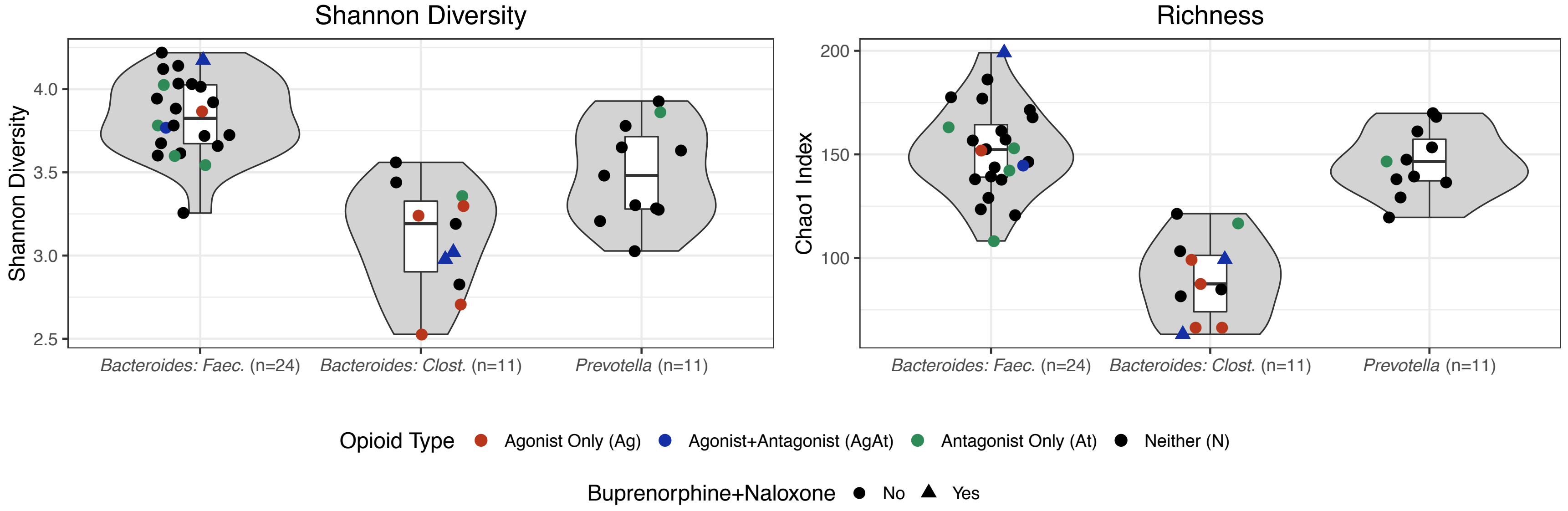

We used Dirichlet multinomial mixture modeling to identify three enterotypes. A hallmark feature of the *Bacteroides: Clost.* group was reduced alpha diversity, here summarized by Shannon diversity (Kruskal Wallis p-value<0.00001) and Chao1 (Kruskal Wallis p-value<0.0001) metrics.

Supplementary Fig. S8 Gut Microbiota Alpha Diversity by Alcohol Use and Fiber Intake among 46 Participants Enrolled from an Outpatient Addiction Treatment Facility, 2016-2017

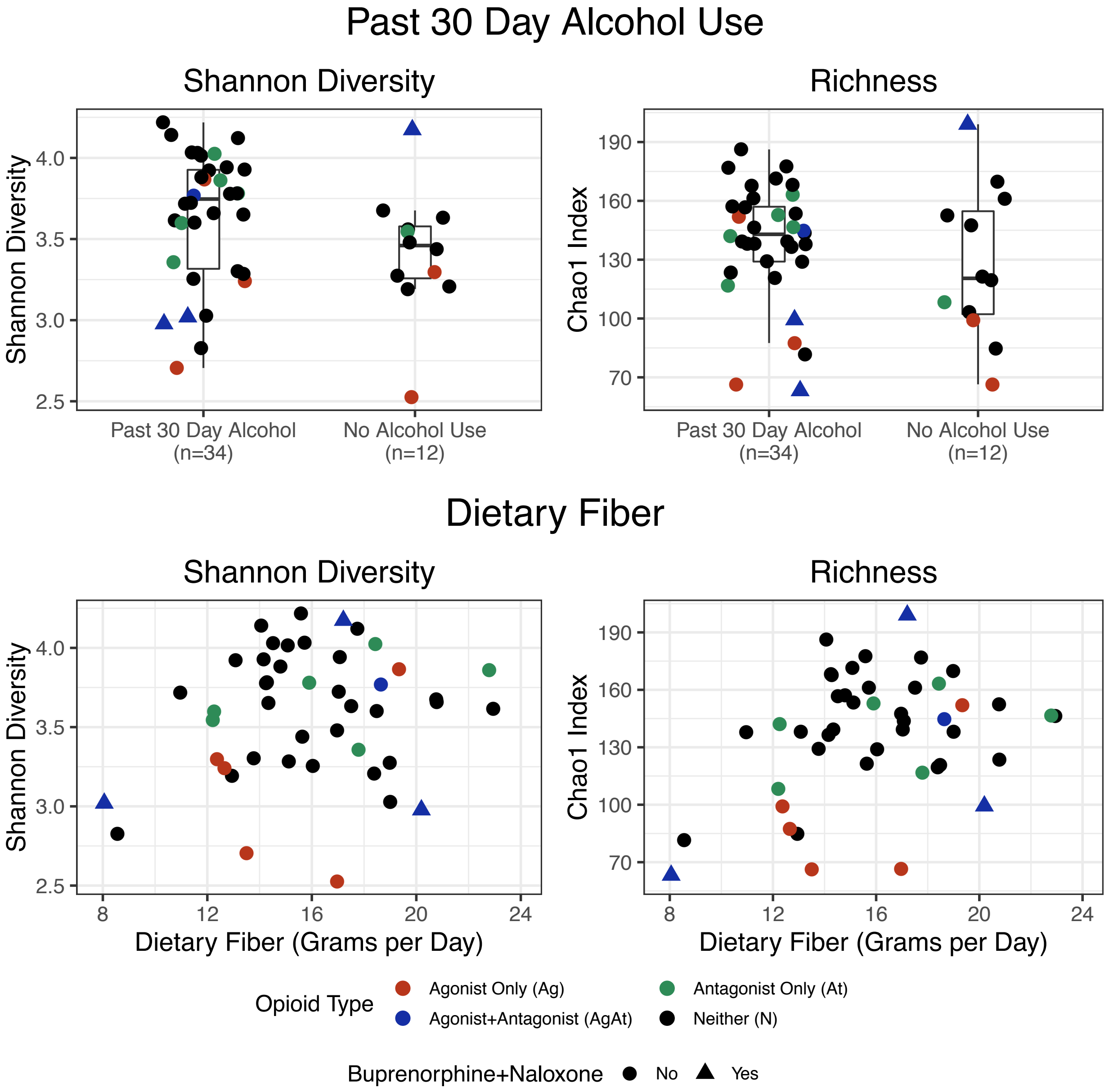

We compared alpha diversity using the Shannon diversity and Chao1 metrics by past 30-day alcohol use (top) and dietary fiber (bottom). Shannon diversity was marginally lower among participants who did not use alcohol in the past 30 days (Wilcoxon rank sum p=0.052). Gut microbiota richness was positively associated with fiber intake and the Pearson correlation (ρ=0.35) between richness and fiber intake reached statistical significance (p=0.02). Other comparisons by alcohol use and fiber intake, including Spearman correlation coefficients for fiber and alpha diversity metrics, did not reach statistical significance.

Supplementary Fig. S9 *De Novo* Assigned Enterotypes by Alcohol Use and Dietary Fiber Intake among 46 Participants Enrolled from an Outpatient Addiction Treatment Facility, 2016-2017

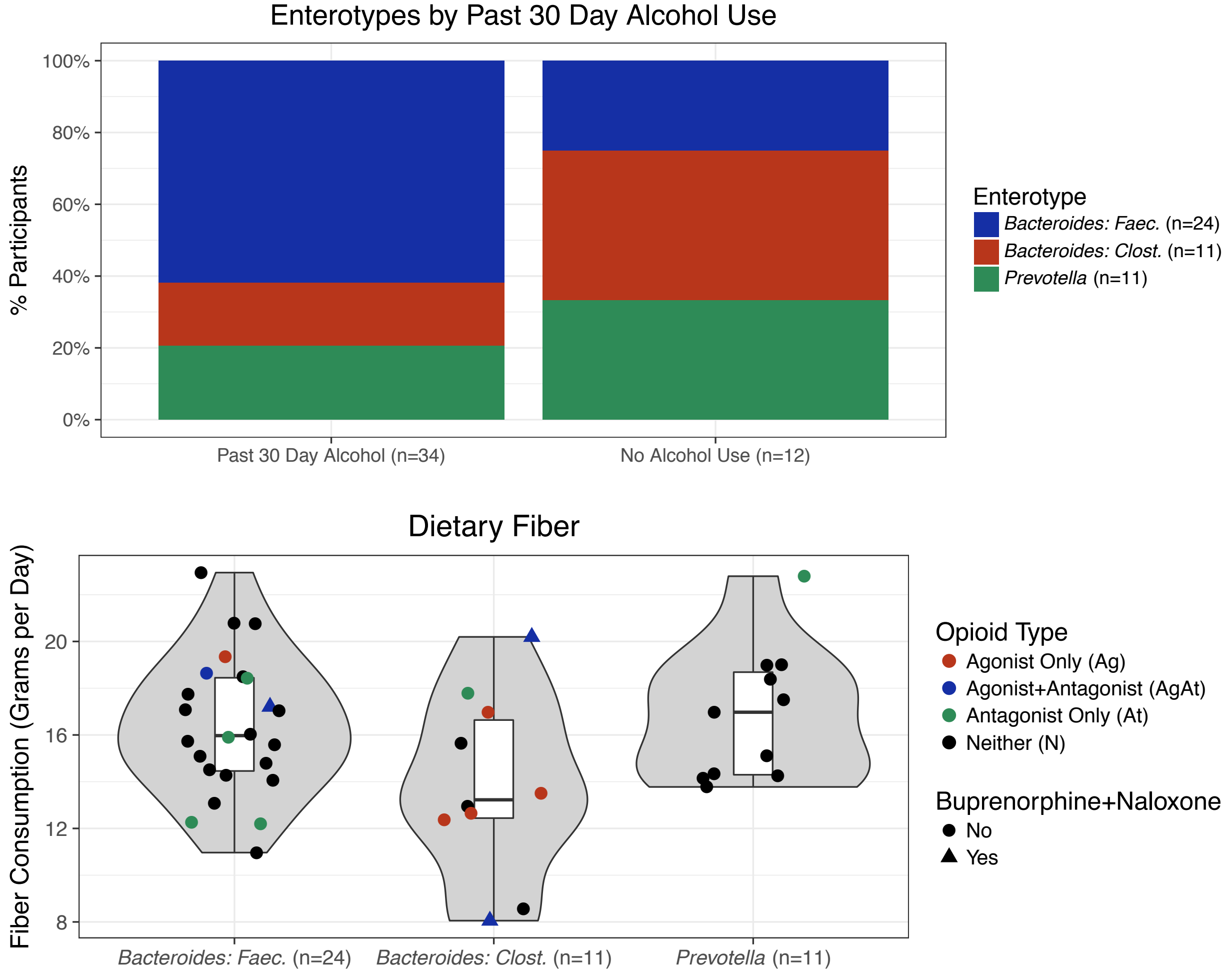

We examined whether enterotype prevalence differed by past 30-day alcohol use (top) or dietary fiber consumption (bottom). The *Bacteroides: Faec* enterotype was marginally more common in people who used alcohol in the past 30 days (Fisher exact p-value=0.11). Participants assigned to *Bacteroides: Clost* had marginally lower fiber intake (Kruskal Wallis p-value=0.12).

Supplementary Table S1 Comparison of Results for Association of Agonist-Antagonist Exposure with Microbiota Features Across Samples Submitted Across 3 Study Visits from 46 Participants Enrolled from an Outpatient Addiction Treatment Facility, 2016-2017

	**Study Visit 1**	**Study Visit 2**	**Study Visit 3**
No. Samples (same as No. participants per visit)	46	44	39
Agonist-Antagonist Groups (No. Participants)			
Agonist Only (Ag)	5	5	5
Agonist+Antagonist (AgAt)	4	4	2
Antagonist (At)	6	4	4
Neither (N)	31	31	28
Alpha Diversity			
Shannon Diversity: Ag vs. N (p-value)	p=0.04 (Fig. 1)	p=0.05	p=0.08
Richness (Chao1): Ag vs. N (p-value)	p=0.008 (Fig. 1)	p=0.01	p=0.01
Shannon Diversity: All Other Agonist-Antagonist Pairwise Comparisons (p-value)	p≥0.05 (Fig. 1)	p≥0.05	p≥0.05
Chao1: All Other Agonist-Antagonist Pairwise Comparisons (p-value)	p≥0.05 (Fig. 1)	p≥0.05	p≥0.05
Beta Diversity	See Fig. S3 (including for legend to read plots at right);<br><br>Conclusion: little visual clustering by agonist-agonist group	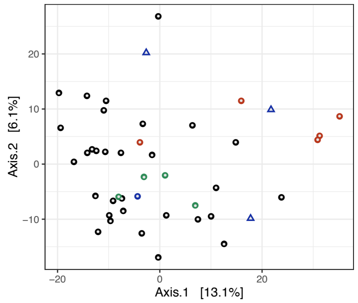<br><br>Conclusion: little visual clustering by agonist-agonist group	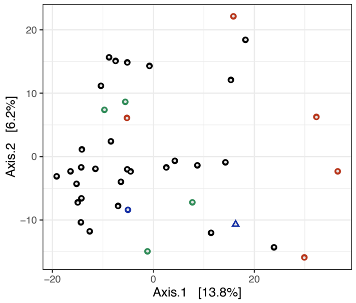<br><br>Conclusion: little visual clustering by agonist-agonist group

De novo Enterotypes*	See Fig. 2<br><br>Enterotype distribution differed in Ag vs. N (p=0.006)<br><br>Other groups: no difference (p≥0.05)<br><br>See Fig. S7: Shannon diversity and richness (Chao1) lowest in *Bacteroides: Clost.* (both p<0.0001)	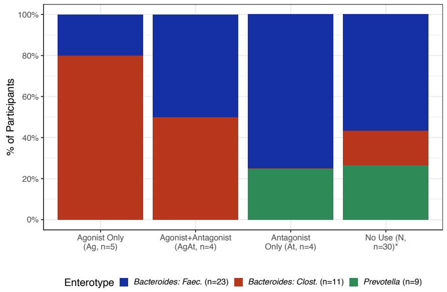<br>Enterotype distribution differed in Ag vs. N (p=0.02)<br><br>Other groups: no difference (p>0.05)<br><br>Shannon diversity and richness (Chao1) lowest in *Bacteroides: Clost.* (both p<0.0001)	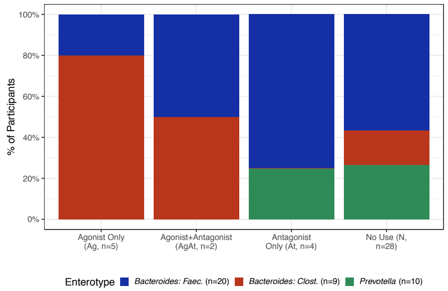<br>Enterotype distribution differed in Ag vs. N (p=0.01)<br><br>Other groups: no difference (p>0.05)<br><br>Shannon diversity and richness (Chao1) lowest in *Bacteroides: Clost.* (both p<0.0001)
Reference-based Enterotypes	42/44 same reference-based and *de novo* assignment (Table 2)<br><br>No *Prevotella* enterotype among Ag or AgAt<br><br>2/46 unable to be assigned reference-based enterotype were assigned *Bacteroides: Clost. de novo* enterotype	34/44 same reference-based and *de novo* assignment<br><br>No *Prevotella* enterotype among Ag, AgAt, or At<br><br>5/44 unable to be assigned reference-based enterotype were *Bacteroides: Clost.* (n=4) and *Prevotella* (n=1) *de novo* enterotype; 1/44 unable to be assigned *de novo* enterotype was assigned *Prevotella* reference-based enterotype	32/39 same reference-based and *de novo* assignment<br><br>No P*revotella* enterotype among Ag, AgAt, or At<br><br>5/39 unable to be assigned reference-based were *Bacteroides: Clost. de novo* enterotype; 1 *Bacteroides: Faec.* was assigned reference-based *Prevotella* enterotype; 1 *Bacteroides: Clost.* assigned Firmicutes reference-based enterotype

Differentially Abundant Genera (ALDEx2)			
Ag vs. N (FDR p-value)†	*Higher in Ag vs. N*<br>• Unclassified Enterobacteriaceae (p = 0.026)<br>• *Lactobacillus* (p = 0.031)<br>• Clostridium_XlVa (p=0.033)<br>• *Faecalicoccus* (p=0.037)<br>• *Anaerostipes* (p=0.040)<br>• *Streptococcus* (p=0.045)<br><br>*Lower in Ag vs. N*<br>• Unclassified Firmicutes (p=0.031)<br>• *Bilophila* (p=0.037)<br>• *Roseburia* (p=0.043)	*Higher in Ag vs. N*<br>• Unclassified Enterobacteriaceae (p = 0.035)<br>• Clostridium_XlVa (p=0.049)<br>• *Bacteroides* (p=0.025)<br>• *Faecalibacterium* (p=0.048)<br><br>*Lower in Ag vs. N*<br>• *Roseburia* (p=0.0018)	*Higher in Ag vs. N*<br>• None (all p≥0.05)<br><br>*Lower in Ag vs. N*<br>• *Roseburia* (p=0.031)
AgAt vs. N (FDR p-value)†	None (all p≥0.05)	None (all p≥0.05)	None (all p≥0.05)
At vs. N (FDR p-value)†	None (all p≥0.05)	None (all p≥0.05)	None (all p≥0.05)

*1 sample from study visit 2 was unable to be assigned a *de novo* enterotype with as the posterior probability of enterotype assignment was <90%.

†Wilcoxon Rank Sum test p-value with Bejamini-Hochberg correction for False Discovery Rate (FDR).

Abbreviations: No.: Number.

References

1. WHO ASSIST Working Group. The Alcohol, Smoking and Substance Involvement Screening Test (ASSIST): development, reliability and feasibility. *Addict. Abingdon Engl.* 97, 1183–1194 (2002).
2. Humeniuk, R. *et al.* Validation of the Alcohol, Smoking And Substance Involvement Screening Test (ASSIST). *Addict. Abingdon Engl.* 103, 1039–1047 (2008).
3. Butler, S. F. *et al.* Development and validation of the Current Opioid Misuse Measure. *Pain* 130, 144–156 (2007).
4. Kroenke, K., Spitzer, R. L. & Williams, J. B. The PHQ-9: validity of a brief depression severity measure. *J. Gen. Intern. Med.* 16, 606–613 (2001).
5. Spitzer, R. L., Kroenke, K., Williams, J. B. W. & Löwe, B. A brief measure for assessing generalized anxiety disorder: the GAD-7. *Arch. Intern. Med.* 166, 1092–1097 (2006).
6. Flannery, B. A., Volpicelli, J. R. & Pettinati, H. M. Psychometric properties of the Penn Alcohol Craving Scale. *Alcohol. Clin. Exp. Res.* 23, 1289–1295 (1999).
7. Flannery, B. A., Poole, S. A., Gallop, R. J. & Volpicelli, J. R. Alcohol craving predicts drinking during treatment: an analysis of three assessment instruments. *J. Stud. Alcohol* 64, 120–126 (2003).
8. Tsui, J. I., Anderson, B. J., Strong, D. R. & Stein, M. D. Craving predicts opioid use in opioid-dependent patients initiating buprenorphine treatment: a longitudinal study. *Am. J. Drug Alcohol Abuse* 40, 163–169 (2014).
9. Costea, P. I. *et al.* Enterotypes in the landscape of gut microbial community composition. *Nat. Microbiol.* 3, 8–16 (2018).

10. Wu, G. D. *et al.* Linking long-term dietary patterns with gut microbial enterotypes. *Science* 334, 105–108 (2011).
11. Vandeputte, D. *et al.* Stool consistency is strongly associated with gut microbiota richness and composition, enterotypes and bacterial growth rates. *Gut* 65, 57–62 (2016).
12. Müller, M., Canfora, E. E. & Blaak, E. E. Gastrointestinal Transit Time, Glucose Homeostasis and Metabolic Health: Modulation by Dietary Fibers. *Nutrients* 10, (2018).
13. National Cancer Institute Division of Cancer Control & Population Sciences, Epidemiology and Genomics Research Program. Data Processing & Scoring Procedures Using Current Methods (Recommended). https://epi.grants.cancer.gov/nhanes/dietscreen/scoring/current/ (2018).
14. National Cancer Institute Division of Cancer Control & Population Sciences, Epidemiology and Genomics Research Program. Dietary Screener Questionnaires (DSQ) in the NHANES 2009-10: DSQ. https://epi.grants.cancer.gov/nhanes/dietscreen/dsq_english.pdf (2018).
15. Feigelson, H. S. *et al.* Feasibility of self-collection of fecal specimens by randomly sampled women for health-related studies of the gut microbiome. *BMC Res. Notes* 7, 204 (2014).
16. Flores, R. *et al.* Collection media and delayed freezing effects on microbial composition of human stool. *Microbiome* 3, 33 (2015).
17. Fu, B. C. *et al.* Characterization of the gut microbiome in epidemiologic studies: the multiethnic cohort experience. *Ann. Epidemiol.* 26, 373–379 (2016).
18. Seekatz, A. M. *et al.* Fecal Microbiota Transplantation Eliminates Clostridium difficile in a Murine Model of Relapsing Disease. *Infect. Immun.* 83, 3838–3846 (2015).
19. Microbial Systems Molecular Biology Laboratory. Internal Protocol for Submitting Samples for DNA Extraction and Illumina 16S rRNA gene sequencing, V4 region. (2017).

20. Kozich, J. J., Westcott, S. L., Baxter, N. T., Highlander, S. K. & Schloss, P. D. Development of a dual-index sequencing strategy and curation pipeline for analyzing amplicon sequence data on the MiSeq Illumina sequencing platform. *Appl. Environ. Microbiol.* AEM.01043-13 (2013) doi:10.1128/AEM.01043-13.
21. Eren, A. M. *et al.* Oligotyping: differentiating between closely related microbial taxa using 16S rRNA gene data. *Methods Ecol. Evol.* 4, 1111–1119 (2013).
22. Berry, M. A. *et al.* Are Oligotypes Meaningful Ecological and Phylogenetic Units? A Case Study of Microcystis in Freshwater Lakes. *Front. Microbiol.* 8, 365 (2017).
23. Wang, Q., Garrity, G. M., Tiedje, J. M. & Cole, J. R. Naive Bayesian classifier for rapid assignment of rRNA sequences into the new bacterial taxonomy. *Appl. Environ. Microbiol.* 73, 5261–5267 (2007).
24. Weiss, S. *et al.* Normalization and microbial differential abundance strategies depend upon data characteristics. *Microbiome* 5, 27 (2017).
25. Knight, R. *et al.* Best practices for analysing microbiomes. *Nat. Rev. Microbiol.* 16, 410–422 (2018).
26. McMurdie, P. J. & Holmes, S. Waste not, want not: why rarefying microbiome data is inadmissible. *PLoS Comput. Biol.* 10, e1003531 (2014).
27. Gloor, G. B., Macklaim, J. M., Pawlowsky-Glahn, V. & Egozcue, J. J. Microbiome Datasets Are Compositional: And This Is Not Optional. *Front. Microbiol.* 8, (2017).
28. Gloor, G. B., Wu, J. R., Pawlowsky-Glahn, V. & Egozcue, J. J. It's all relative: analyzing microbiome data as compositions. *Ann. Epidemiol.* 26, 322–329 (2016).
29. Aitchison, J. The Statistical Analysis of Compositional Data. *J. R. Stat. Soc. Ser. B Methodol.* 44, 139–177 (1982).

30. Holmes, I., Harris, K. & Quince, C. Dirichlet multinomial mixtures: generative models for microbial metagenomics. *PloS One* 7, e30126 (2012).

31. Fernandes, A. D., Macklaim, J. M., Linn, T. G., Reid, G. & Gloor, G. B. ANOVA-like differential expression (ALDEx) analysis for mixed population RNA-Seq. *PloS One* 8, e67019 (2013).

32. Arumugam, M. *et al.* Enterotypes of the human gut microbiome. *Nature* 473, 174–180 (2011).
